# MamO Is a Repurposed Serine Protease that Promotes Magnetite Biomineralization through Direct Transition Metal Binding in Magnetotactic Bacteria

**DOI:** 10.1371/journal.pbio.1002402

**Published:** 2016-03-16

**Authors:** David M. Hershey, Xuefeng Ren, Ryan A. Melnyk, Patrick J. Browne, Ertan Ozyamak, Stephanie R. Jones, Michelle C. Y. Chang, James H. Hurley, Arash Komeili

**Affiliations:** 1 Department of Plant and Microbial Biology, University of California, Berkeley, California, United States of America; 2 Department of Molecular and Cellular Biology, University of California, Berkeley, California, United States of America; 3 California Institute for Quantitative Biosciences, University of California, Berkeley, California, United States of America; 4 Physical Biosciences Division, Lawrence Berkeley National Laboratory, Berkeley, California, United States of America; 5 Department of Chemistry, University of California, Berkeley, California, United States of America; 6 Life Sciences Division, Lawrence Berkeley National Laboratory, Berkeley, California, United States of America; Rutgers University-Robert Wood Johnson Medical School, UNITED STATES

## Abstract

Many living organisms transform inorganic atoms into highly ordered crystalline materials. An elegant example of such biomineralization processes is the production of nano-scale magnetic crystals in magnetotactic bacteria. Previous studies implicated the involvement of two putative serine proteases, MamE and MamO, during the early stages of magnetite formation in *Magnetospirillum magneticum* AMB-1. Here, using genetic analysis and X-ray crystallography, we show that MamO has a degenerate active site, rendering it incapable of protease activity. Instead, MamO promotes magnetosome formation through two genetically distinct, noncatalytic activities: activation of MamE-dependent proteolysis of biomineralization factors and direct binding to transition metal ions. By solving the structure of the protease domain bound to a metal ion, we identify a surface-exposed di-histidine motif in MamO that contributes to metal binding and show that it is required to initiate biomineralization in vivo. Finally, we find that pseudoproteases are widespread in magnetotactic bacteria and that they have evolved independently in three separate taxa. Our results highlight the versatility of protein scaffolds in accommodating new biochemical activities and provide unprecedented insight into the earliest stages of biomineralization.

## Abstract 2

Genetic, structural, and phylogenetic analyses reveal that MamO is a degenerate descendant of serine proteases that has acquired a new function in promoting the synthesis of magnetic iron oxide particles in bacteria.

## Author Summary

Biomineralization is an ancient and ubiquitous process by which organisms assemble crystalline materials for their own benefit. The ability to precisely organize inorganic atoms into crystals with intricate shapes demonstrates a level of control over nanoparticle synthesis that has fascinated biologists for generations. We have been studying how a group of microorganisms, called magnetotactic bacteria, synthesizes iron-based crystals that are used for navigation along magnetic fields. Here, we characterize a protein called MamO that helps to initiate the formation of a magnetic mineral called magnetite in cells of the magnetotactic bacterium *Magentospirillum magneticum* AMB-1. Although predicted to be a trypsin-like protease, we show that MamO has lost its ancestral catalytic activity and instead gained a new function as a metal-binding scaffold. By solving its structure, we discovered how MamO binds to transition metal atoms and show that this activity is required to crystalize magnetite within cells. Surprisingly, we find that similar repurposed trypsin-like proteases have evolved independently in all three major magnetotactic groups, outlining a fascinating case of convergent evolution. The unique evolutionary history of MamO demonstrates that existing protein scaffolds can be modified to provide new functions and contributes to our understanding of how cells build transition metal-based minerals.

## Introduction

Biomineralization is the widespread phenomenon by which living organisms transform inorganic atoms into highly ordered, crystalline structures. Controlling the size and shape of such materials requires specialized protein machinery that can define the nano-scale trajectory of crystal growth [1]. Incorporating biochemical principles uncovered from studying biomineralization has the potential to revolutionize the design and synthesis of nanomaterials in vitro [2]. In addition to the well-known examples of tooth, bone, and shell production by multicellular eukaryotes, a number of bacteria have the ability to biomineralize small magnetic crystals within subcellular compartments called magnetosomes [3,4]. These particles allow the cells to passively align in the earth’s magnetic field, facilitating the search for their preferred oxygen environments [5]. Although these magnetotactic bacteria have drawn longstanding interest due to their ability to manipulate transition metals, the biochemical details of how they transform iron into magnetite (Fe_3_O_4_) remain poorly understood.

Magnetotactic organisms are phylogentically diverse. Nearly all isolates come from the α-, δ-, or γ- classes of *Proteobacteria*, but representatives from the *Nitrospirae* and *Omnitrophica* phyla have recently been identified [6]. The genes responsible for making magnetosomes are often contained in a genomic region called the magnetosome island (MAI) [7–11]. Comparative genomic and phylogenetic studies have identified a set of core genes that appears to have been assembled a single time and inherited vertically, indicating that magnetosome formation likely predates the divergence of the *Proteobacteria* [12,13]. The MAI seems to have formed by incorporating elements from other cellular processes, as the majority of the core factors have homology to ancient and widespread protein domains [14,15]. Uncovering the biochemical functions encoded in the MAI in relation to its evolutionary history provides a unique opportunity to understand how new cellular processes evolve.

Due to the availability of genetic systems, α*-Proteobacteria* such as *Magnetospirillum magneticum* AMB-1 are used as models for studying the molecular biology of magnetosome formation [16]. AMB-1 contains 15–20 magnetite crystals, each formed within a cytoplasmic membrane invagination and organized in a chain spanning the length of the cell [17,18]. By making deletions within the MAI and characterizing the ultrastructure of the mutant cells, specific genes have been assigned roles in various stages of magnetosome formation [10,11,19,20]. Genes whose deletions produced empty magnetosome compartments or compartments with abnormally small magnetite crystals were termed biomineralization factors. It is the proteins encoded by these genes that hold the secrets of how magnetotactic organisms interface with solid magnetite.

**Fig 1:**
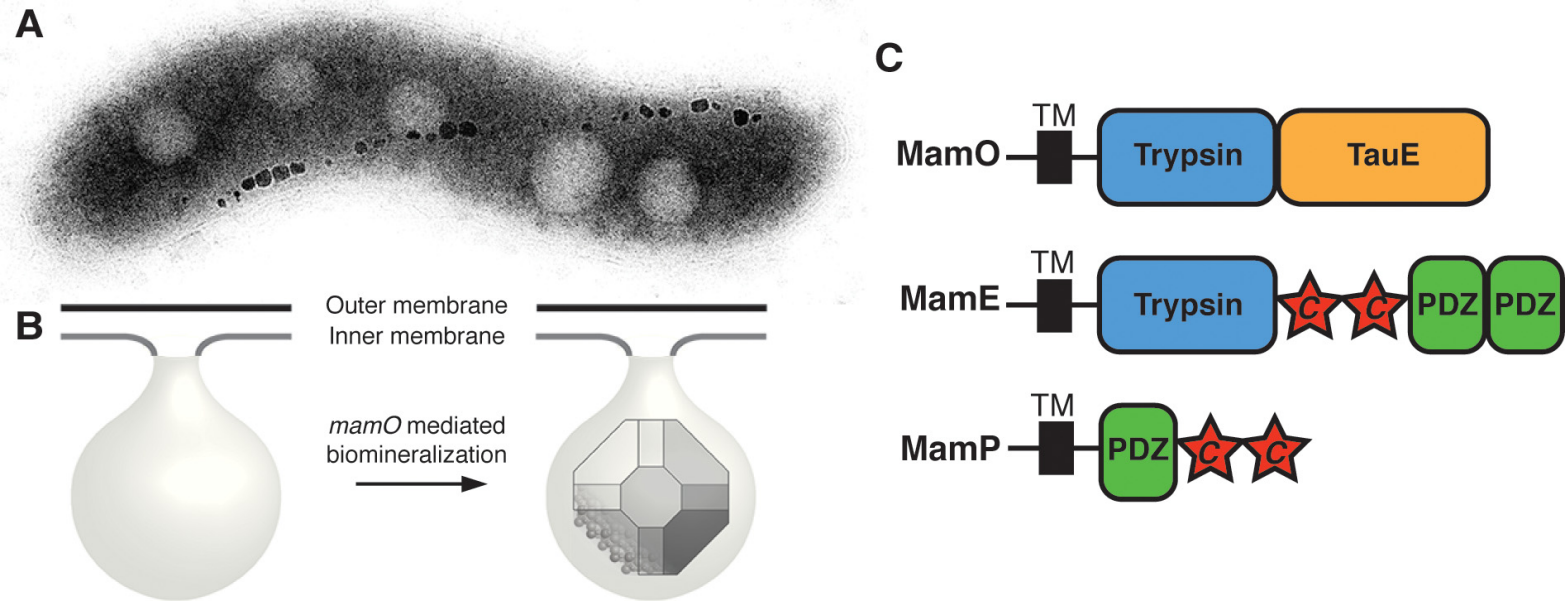
MamO promotes the nucleation of magnetosome crystals. (**A**) TEM micrograph of a wild-type AMB-1 cell. The electron-dense particles make up a magnetosome chain. (**B**) Cellular organization of the magnetosome compartment. MamO promotes the nucleation of magnetite within inner membrane invaginations. (**C**) Domain structure of the three biomineralization factors discussed in the text. “*c*” represents a CXXCH *c*-type cytochrome motif.

Two genes in the MAI, *mamE* and *mamO*, are homologous to the HtrA proteases, a ubiquitous family of trypsin-like enzymes that functions using His-Asp-Ser catalytic triads [21]. An additional pair of genes, called *limE* and *limO*, with homology to the protease domains of *mamE* and *mamO*, exists in a secondary genomic region termed R9 [10]. Disrupting *mamE* or *mamO* causes cells to produce empty magnetosome membranes, but removing R9 has no effect, showing that *mamE* and *mamO* are required for biomineralization and that *limE* and *limO* are not (Fig 1) [10,22]. Adding variants of *mamE* or *mamO* with all three predicted active site residues mutated to alanine could not restore normal biomineralization in the Δ*mamO*Δ*R9* or Δ*mamE*Δ*R9* strains but complemented single Δ*mamO* or Δ*mamE* deletions [23]. These genetic analyses show that *mamE* and *mamO* are required for the initiation of magnetite biomineralization. Furthermore, *limE* and *limO* are partially redundant in that they can cross-complement the active site-dependent crystal maturation defects of their respective orthologs.

Here, we use a combination of in vivo and in vitro approaches to reveal an unexpected dual role for MamO. It promotes MamE-dependent proteolysis of three biomineralization factors through the use of its C-terminal transporter domain. Separately, the protease domain has lost the ability to carry out catalysis and has instead been repurposed to bind transition metal ions. Two surface-exposed histidine residues that contribute to this metal-binding function are required for initiating magnetite biosynthesis in vivo. Bioinformatic analysis shows that similar pseudoproteases evolved independently in the three major taxa of magnetotactic organisms, highlighting a unique evolutionary mechanism behind microbial nanoparticle synthesis.

## Results

### The Catalytic Triad in MamO Is Not Required for Magnetite Nucleation

**Fig 2:**
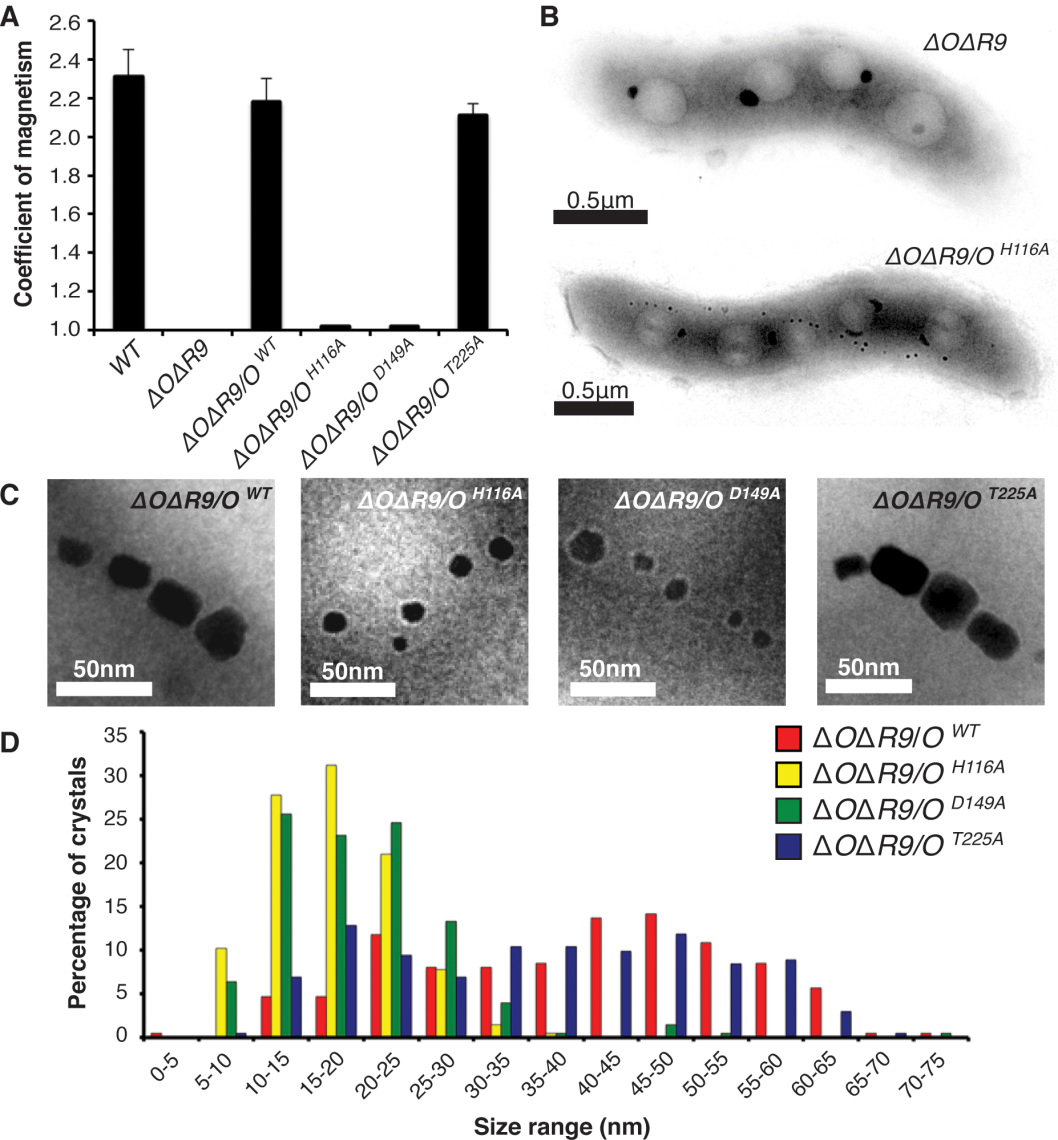
Dissection of the MamO catalytic triad. (**A**) Magnetic response of cultures with the indicated *mamO* alleles. The coefficient of magnetism is an optical density-based method assessing cells’ ability to turn in a magnetic field. Biological replicates represent independent cultures of each strain. Each measurement represents the average and standard deviation from three independent experiments. (**B**) Transmission electron microscopy (TEM) of whole AMB-1 cells with the indicated genetic backgrounds. (**C**) Magnetite crystals from cells with the indicated *mamO* alleles. (**D**) Crystal size distributions for the indicated *mamO* alleles as assessed by TEM. *n* > 200 for each strain.

Trypsin-like proteases utilize a histidine–aspartate pair to deprotonate the hydroxyl group of a serine residue, providing a nucleophile for catalysis. MamO is unusual in that it contains a threonine instead of serine as the predicted nucleophile. To further clarify the role of MamO’s unusual active site, we focused our initial efforts on assessing the contribution of each putative catalytic residue to biomineralization. We performed these initial studies using the Δ*mamO*Δ*R9* strain (referred to as Δ*O*Δ*R9*) to avoid cross-complementation from *limO*. Consistent with our previous findings, individual H116A and D149A mutations in MamO severely reduced the cells’ ability to turn in a magnetic field (Fig 2A). Surprisingly, a T225A mutation had no defects in magnetic response (Fig 2A). Using transmission electron microscopy (TEM), we confirmed that *mamO* ^*H116A*^ and *mamO* ^*D149A*^ cells have small magnetite crystals while *mamO* ^*T225A*^ crystals are indistinguishable from wild-type, mirroring the bulk magnetic response measurements (Fig 2B–2D).

Given that the predicted nucleophile is dispensable for magnetosome formation, we found it curious that the other two catalytic triad mutations disrupted crystal maturation. Upon further examination, we found that the phenotypes associated with the *mamO* ^*H116A*^ and *mamO* ^*D149A*^ alleles were actually temperature-dependent. Growing wild-type AMB-1 at room temperature instead of the standard 30°C did not dramatically alter the magnetic response. However, both the *mamO* ^*H116A*^ and *mamO* ^*D149A*^ alleles displayed improved complementation at the lower temperature. In particular, the *mamO* ^*D149A*^ mutant restored a nearly wild-type magnetic response to the Δ*O*Δ*R9* strain under these conditions (S1 Fig). Our results suggest that protease activity from MamO is not required for biomineralization. None of the three predicted catalytic residues is required for magnetite nucleation, and, although two of the three contribute to crystal maturation, these effects are conditional, suggesting that they are not central to the biomineralization process.

### Proteolytic Processing of Biomineralization Factors

**Fig 3:**
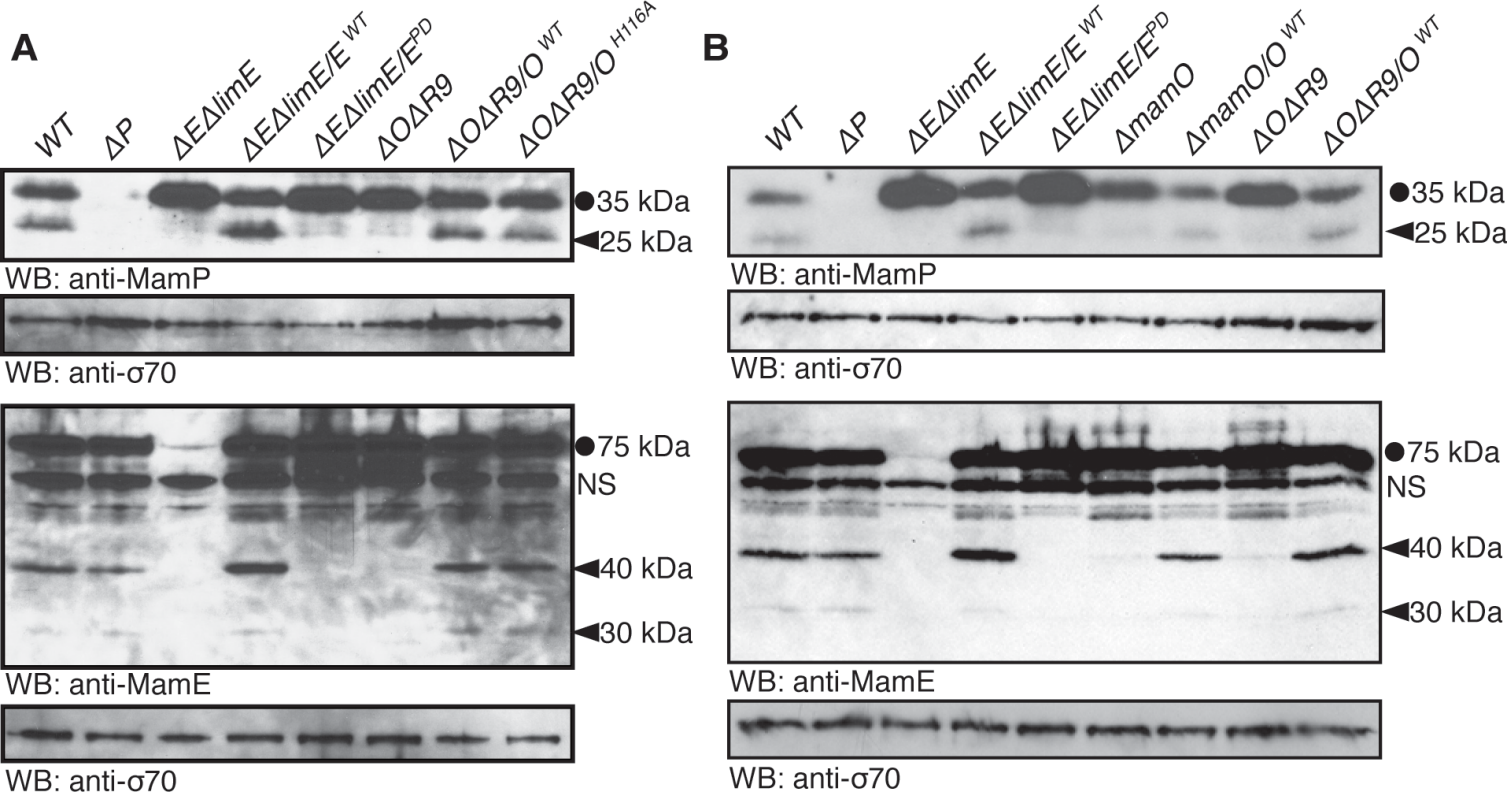
Genetic requirements for proteolytic processing of biomineralization factors. Full-length proteins are marked with a circle and proteolytic fragments with a carat. *mamE* ^*PD*^ refers to the previously described allele with all three catalytic triad residues mutated to alanine. A nonspecific interaction with the anti-MamE antibody is marked with “NS.” (**A**) Proteolysis of MamE and MamP depends on both MamE and MamO, The MamE active site is required, but the MamO active site is dispensable. (**B**) Efficient proteolysis of MamE and MamP requires the TauE domain of MamO. In both the Δ*O*Δ*R9* strain and the Δ*mamO* strain (which contains *limO*), proteolysis of the two targets is minimal.

During the course of our experiments, we examined the MamO variants by western blotting. Although no changes in overall protein abundance were present, we noticed that each MamO variant was proteolytically processed, having both a full-length and shorter form (S2 Fig). This finding led us to examine whether other magnetosome proteins are similarly proteolyzed. We found that MamE and another biomineralization factor, MamP, are also proteolytic targets in AMB-1 cells by using antibodies targeted to each protein (Fig 3A). MamP is a *c*-type cytochrome, and its iron oxidase activity is required for the proper maturation of magnetite crystals [24,25]. Since these three biomineralization factors exist in both full-length and shorter forms, it is likely that proteolytic maturation plays a role in their function.

To examine the potential involvement of *mamE* and *mamO* in promoting these proteolytic events, we analyzed processing of MamO, MamE, and MamP in various genetic backgrounds. In both the Δ*O*Δ*R9* and Δ*E*Δ*limE* strains, we observed only the full-length form of MamP. Similar analyses showed that MamO is required for the proteolysis of MamE and that MamE is required for the proteolysis of MamO. Thus, processing of each target requires both putative proteases (Figs 3A and S2A). Addition of the *mamE* ^*WT*^ allele restored processing of each target in the Δ*E*Δ*limE* strain, but the catalytically inactive form (*mamE* ^*PD*^) did not, suggesting that MamE participates in proteolysis directly. Surprisingly, both the *mamO* ^*WT*^ and *mamO* ^*H116A*^ alleles restored processing in the Δ*O*Δ*R9* strain (Figs 3A and S2). Therefore, the presence of MamO, but not its catalytic triad, appears to be required to promote the activity of MamE. While these results strongly suggest that MamE directly cleaves all three biomineralization factors, we cannot rule out the possibility that its activity contributes to a more complex targeting process.

Given that HtrA proteases are often regulated by the formation of higher order oligomers, an attractive model could be that MamO activates MamE through an interaction involving both protease domains. To test this idea, we exploited the partial redundancy from genes in the R9 region. In addition to its N-terminal protease domain, MamO has a predicted TauE-like transporter domain on its C-terminus. *limO*, the partial duplication of *mamO* in R9, is 98% identical to the protease domain of *mamO* but does not contain a C-terminal TauE domain (S3 Fig). We confirmed that alleles with individual point mutations in the protease domain that reduce bioimineralization in the Δ*O*Δ*R9* background do not have defects in the Δ*mamO* strain, reinforcing that LimO is a functionally redundant copy of the MamO protease domain (S3 Fig). In contrast, processing of MamE and MamP is disrupted in both the Δ*mamO* and Δ*O*Δ*R9* strains, showing that a functional protease domain is insufficient to activate MamE and that activation requires the TauE domain (Fig 3B).

### Structural Analysis of the MamO Protease Domain

In the trypsin family, loop L1 contains both the nucleophilic serine and oxyanion hole, required for creating the acyl-enzyme intermediate and stabilizing the oxyanion, respectively (S4 Fig) [26]. In addition to the threonine substitution, the entire L1 loop in MamO differs significantly from the trypsin family consensus, once again suggesting that MamO might not be capable of protease activity (S4 Fig). To explore this possibility further, we determined the crystal structure of the protease domain (S1 Table). It crystallizes as a monomer with the chymotrypsin fold and the catalytic residues properly placed (S4 Fig).

**Fig 4:**
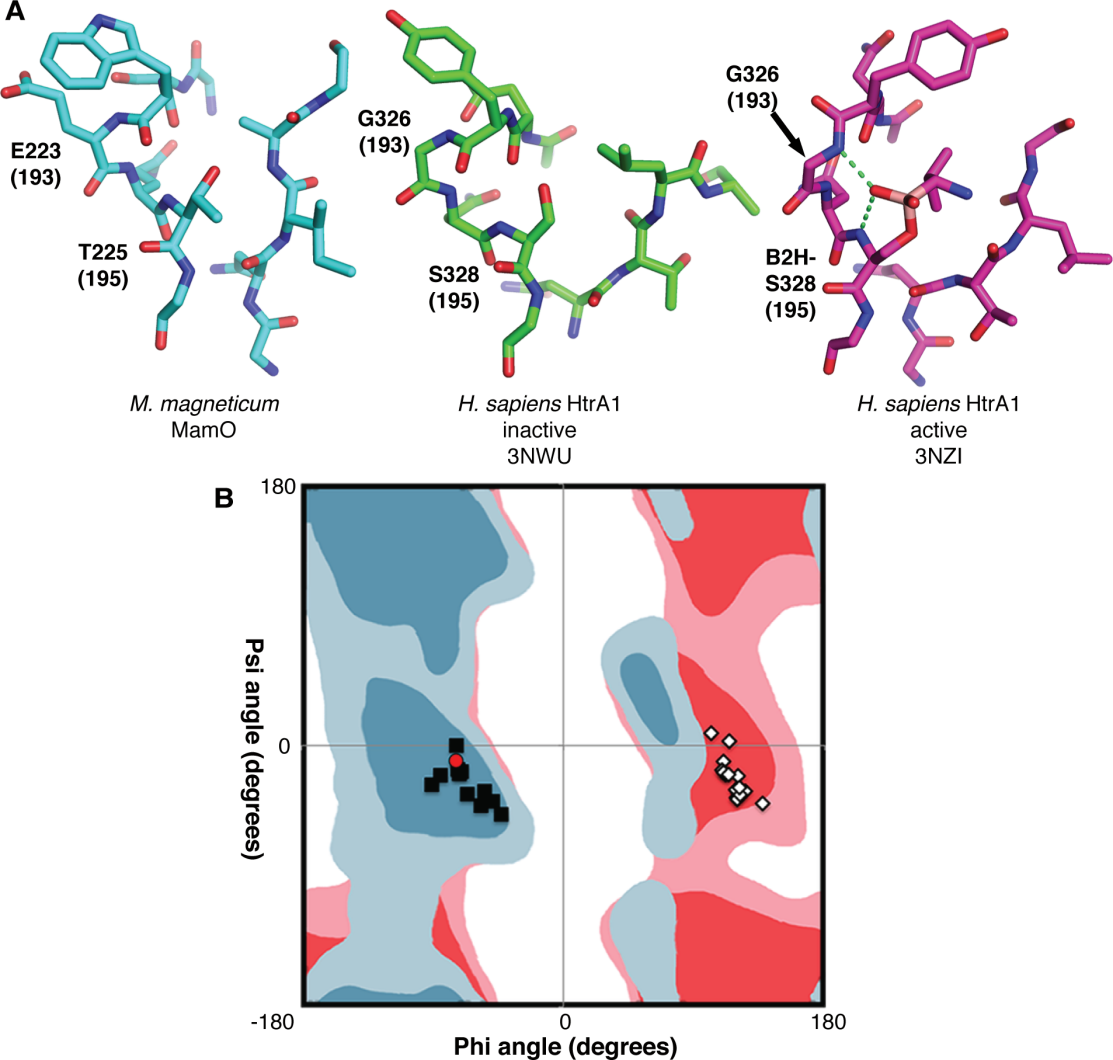
Steric constraints on the MamO active site. (**A**) Comparison of active site structures, showing MamO in the inactive conformation. Each residue’s chymotrypsin numbering position is in parentheses. Dashes represent hydrogen bonds contributed by the oxyanion hole. (**B**) Ramachandran plot showing favored (dark shades) and allowed (light shades) geometries for nonglycine residues (blue) and glycine (red). The configuration at residue 193 for a set of trypsin-like structures is plotted. Black squares: inactive conformation; white diamonds: active conformation; red circle: MamO. The active conformation is disallowed for MamO.

Loop L1 of MamO adopts the inactive conformation seen in many HtrA proteases in which the main chain carbonyl of residue 192 (chymotrypsin numbering; W222 in MamO) prevents access to the oxyanion hole (Fig 4A). In other HtrAs, the inactive form is thought to be in equilibrium with an active conformation in which the main chain flips approximately 180°, opening the oxyanion hole (Fig 4A) [27,28]. Switching to the active state forces residue 193 into a configuration that is strongly disfavored for nonglycine residues. Although glycine is highly conserved at position 193 in the trypsin family and is critical for catalysis, MamO contains a glutamate (E223) at this position [29]. Therefore, the active configuration of MamO would contain a strong steric clash between the E223 side chain and the main chain carbonyl of W222.

To illustrate this steric constraint, we examined a set of trypsin-like protease structures and analyzed the configuration of residue 193. We plotted ϕ and Ψ values for this position on a Ramachandran plot along with the favored and allowed geometries for glycine and nonglycine residues (S2 Table) [30]. As expected, the main-chain torsion angles at position 193 form two groups, distinguished by an approximately180° shift in ϕ angle. The groups correspond to active and inactive forms of loop L1. The active configuration is strongly disfavored unless glycine occupies position 193, indicating that the E223 side-chain in MamO likely prevents the formation of an oxyanion hole (Fig 4B).

**Fig 5:**
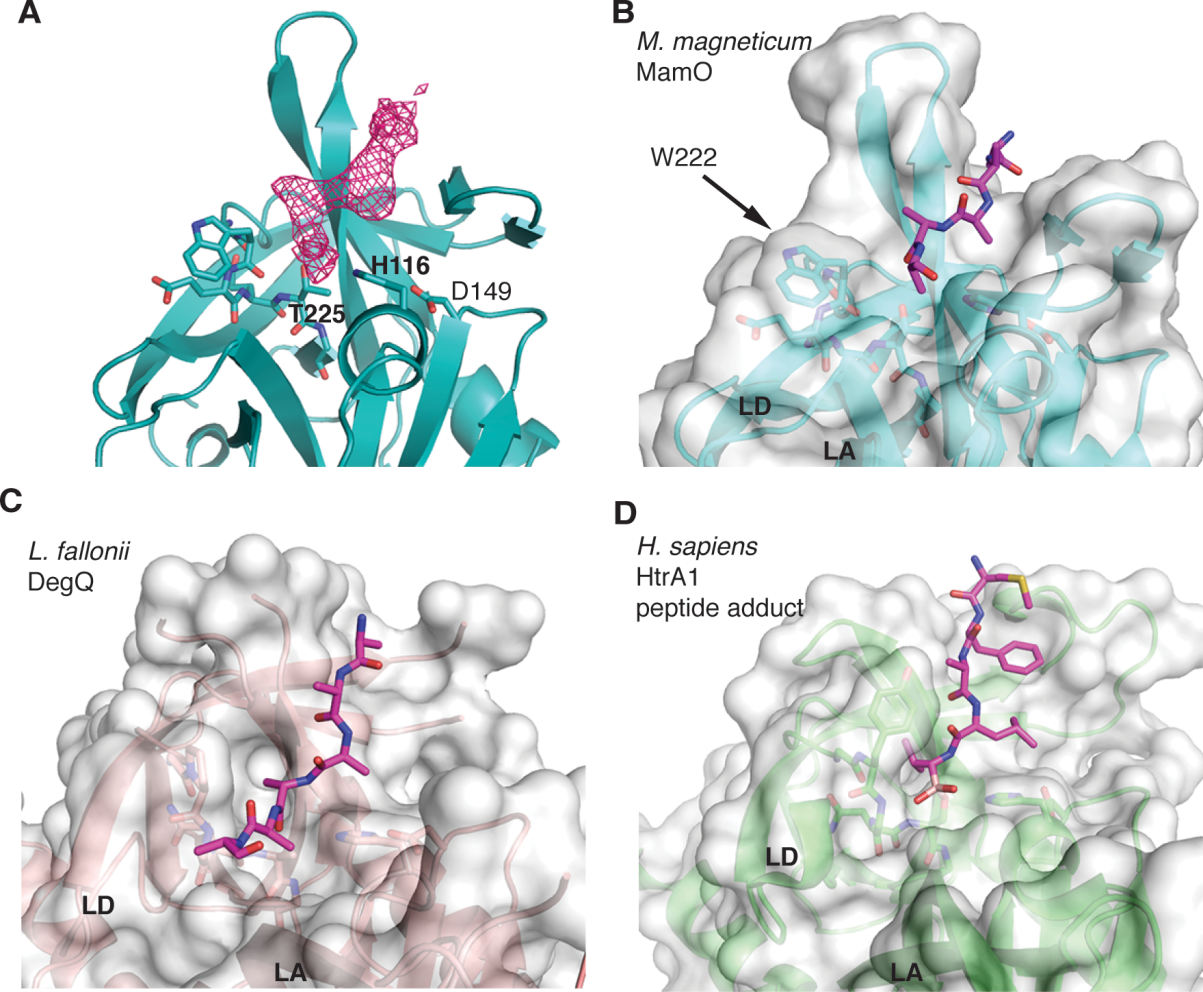
W222 in MamO forces the bound peptide away from the active site. (**A**) *F*_*o*_ *−F*_*c*_ omit map contoured at 3σ showing missing density for the peptide in the MamO crystal structure. (**B-D**) Comparison of peptide binding pockets in HtrA proteases. W222 in MamO blocks the normal exit path between loops LA and LD and pushes the peptide away from the active site. PDB codes for each panel are as follows B: 5HM9; C: 3PV3; D: 3NZI. Loops LA and LD are marked.

While refining the MamO structure, we observed a peptide bound near the predicted peptide-binding groove (Fig 5A). We suspect its source is the flexible N-terminal region from a neighboring MamO in the crystal that is not built into the model. However, due to the modest resolution, we cannot confirm the sequence. Despite this uncertainty, the peptide has an interesting mode of binding. Similar to other trypsin-like proteases, the peptide enters the binding cleft parallel to loop L2, splitting the two β-barrels of the chymotrypsin fold. However, the bulky side-chain of W222 in MamO seems to block the exit path between loops LA and LD, forcing the peptide away from the catalytic center (Fig 5B–5D). Overall, the structural features of the L1 loop appear incompatible with protease activity: E223 provides an energetic barrier to catalysis while W222 serves as a physical barrier to productive substrate binding.

### Direct Transition Metal Binding by MamO

Given the results of the genetic studies and structural analysis, we conclude that MamO does not act as a protease during biomineralization. This forced us to consider the possibility that the protease domain promotes magnetite formation using a function not predicted from its primary sequence. While purifying MamO, we serendipitously observed that it consistently bound to immobilized metal affinity columns. Knowing that mutations in MamO cause defects in AMB-1’s ability to transform iron, a metal, into magnetite, we speculated that direct metal binding played a role in biomineralization.

**Fig 6:**
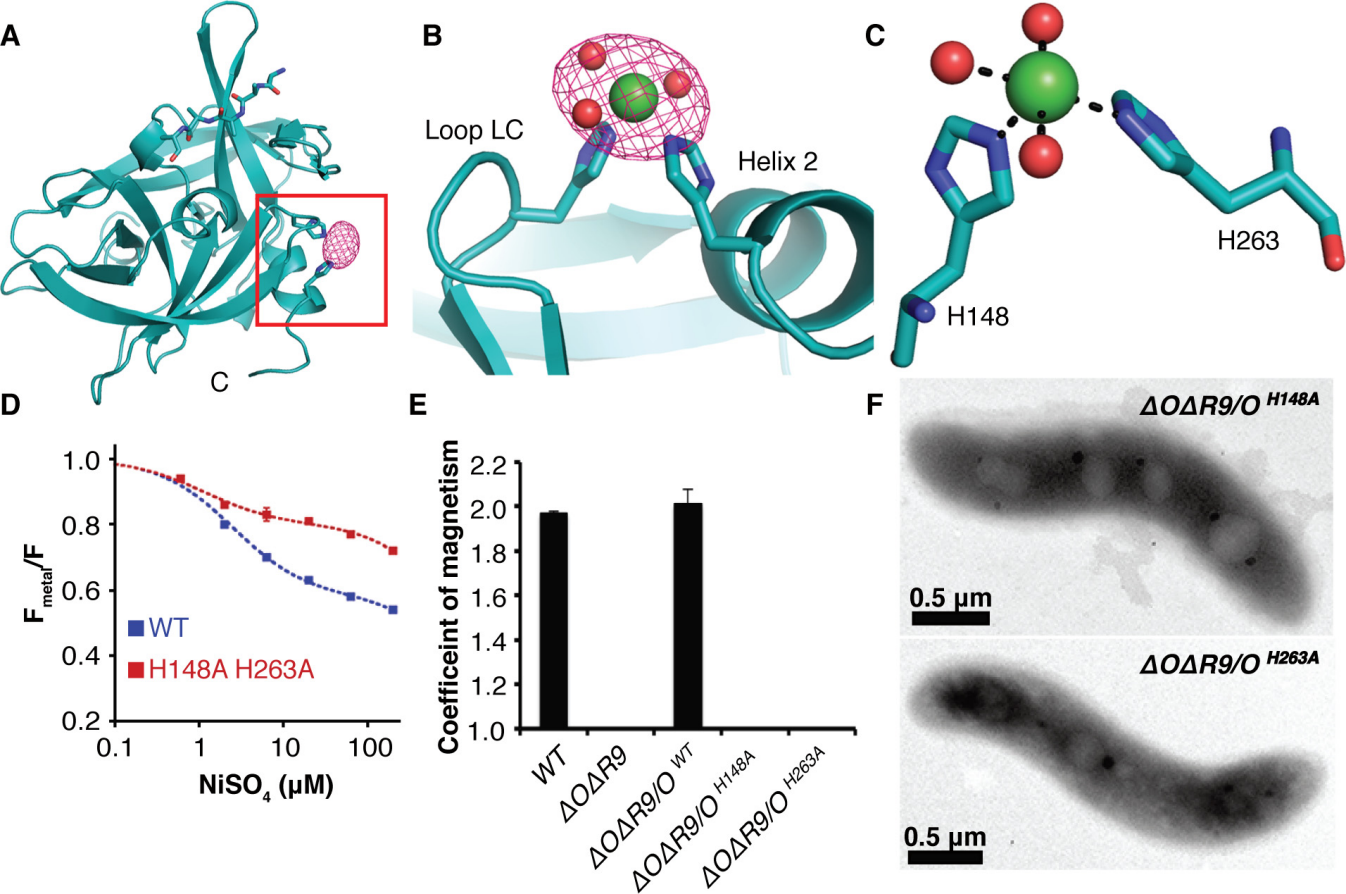
Characterization of the MamO metal binding site. (**A**) Anomalous map contoured at 5σ showing the placement of Ni^2+^ in MamO. (**B**) Ni^2+^ ion bound between loop LD and helix 2. (**C**) H148, H263, and three ordered water molecules participate in metal binding. (**D**) Transition metal ion Förster Resonance Energy Transfer (tmFRET) analysis of Ni^2+^ binding by MamO labeled at Q258C. Each measurement represents the average from four replicates. Error bars represent the standard deviation of the replicates. (**E**) Magnetic response of strains with disrupted metal coordination sites. Error bars represent the standard deviation from three cultures. Each measurement represents the average of three biological replicates. Error bars represent the standard deviation of the replicates. (**F**) TEM analysis showing that the *mamO* ^*H148A*^ and *mamO* ^*H263A*^ strains lack detectable minerals.

Although the MamO crystals grow in acidic conditions that disfavor metal binding, they could be soaked at pH 8.0 without affecting diffraction. We solved another structure of the protease domain using crystals soaked in NiCl_2_ at pH 8.0 and identified a metal binding site. Overall, the conformation of the protease domain is highly similar to the original structure (root-mean-square deviation of 0.17 Å over 184 residues), and it contains the unidentified peptide. Additionally, a single Ni^2+^ ion binds between loop LC and helix 2, with H148 and H263 directly coordinating the metal (Fig 6A–6C). We confirmed the placement of the ligand at this site using single wavelength anomalous dispersion (SAD) data collected at the Ni absorption edge (Fig 6A and 6B). MamO’s di-histidine motif is highly reminiscent of the Zn^2+^ binding site in another trypsin-like protease, kallikrein-3, in which Zn^2+^attenuates protease activity by altering the position of catalytic triad residues H57 and D102 (S5 Fig) [31,32].

To confirm the putative metal binding site from the MamO structure, we used transition metal ion Förster Resonance Energy Transfer (tmFRET) to assay binding in solution [33]. This technique measures fluorescence quenching of a cysteine-conjugated fluorophore by a metal bound at a nearby site. Guided by the structure, we targeted Q258 because of its optimal geometry relative to the metal. Adding a number of transition metals, including iron, to a purified Q258C mutant protease domain that was labeled with fluorescein-5-maleimide caused strong fluorescence quenching (S6 and S7 Figs). Although we expect Fe^2+^ to be the physiological ligand, its propensity to oxidize in ambient atmosphere added significant error to the measurements. Instead, the quenching properties and resistance to oxidation of Ni^2+^ were most suitable for detailed analysis. MamO bound to Ni^2+^ with 2.5 μM affinity, compared to 1.1 μM in an H148A/H263A mutant. Additionally, the FRET efficiency was significantly lower in the mutant protein, demonstrating that disrupting these residues changes the metal binding properties of MamO (Figs 6D and S7).

Although the H148A/H263A mutant displays altered behavior in the tmFRET assay, it retains the ability to bind metals (S7 Fig). We could not identify any other metal ions in our Ni^2+^ soaked crystals, but the two histidines we identified did not appear to be the only metal binding residues in MamO. Consistent with this, both the wild-type and H148A/H263A forms of MamO bound Ni-NTA resin, confirming that, despite its altered binding geometry, the mutant still binds to metal (S7 Fig). Taken together, our biochemical and structural investigations show that MamO binds to transition metal ions using H148 and H263, but that metal binding is not restricted to this motif.

Because disrupting H148 and H263 altered the metal binding behavior in vitro, we predicted that these residues were important for MamO-dependent biomineralization. Indeed, Δ*O*Δ*R9* strains with *mamO* ^*H148A*^ or *mamO* ^*H263A*^ alleles had no magnetic response and failed to produce electron-dense particles, implying that proper metal binding is required to initiate biomineralization (Fig 6E and 6F). Both mutants displayed normal stability and proteolytic processing, and the biomineralization defects were not conditional, as lowering the growth temperature did not allow for a magnetic response (S1 and S2 Figs). Additionally, both alleles restored a magnetic response in the single Δ*mamO* background, showing that *limO* can provide the required metal binding activity independent of cotranslation with the TauE domain (S3 Fig). These are the most disruptive point mutations we have observed in MamO, and they completely recapitulate the phenotype of a *mamO* deletion. We conclude that H148 and H263 contribute to a metal binding function that is required for magnetite nucleation in vivo.

### Convergent Evolution of Pseudoproteases in Magnetotactic Bacteria

**Fig 7:**
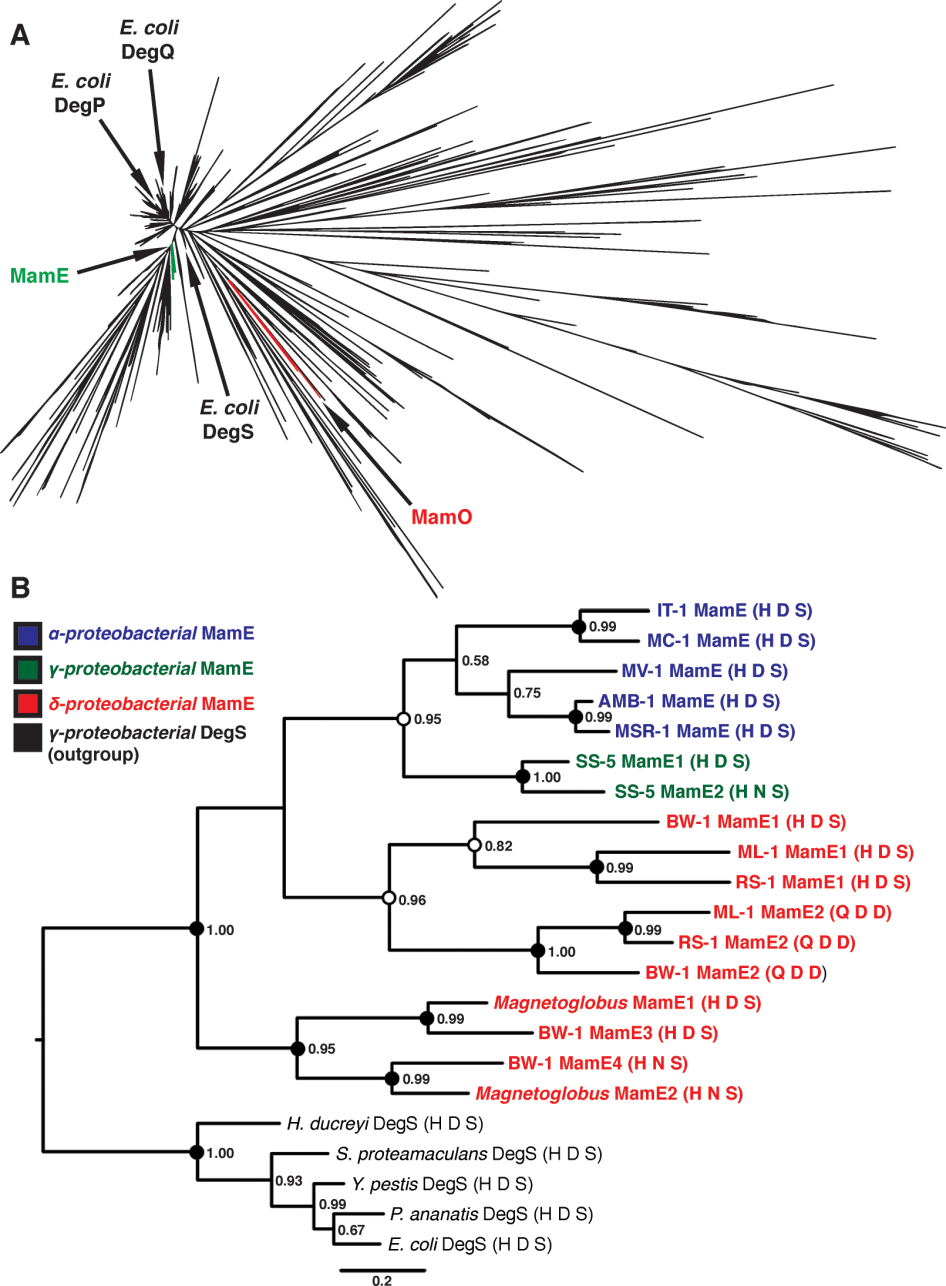
Phylogenetic analysis of magnetotactic trypsin-like proteins. (**A**) Phylogeny of the “Deg” branch created from the trimmed Trypsin-2 alignment. MamE and MamO clades and the three *Escherichia coli* HtrA proteases are marked. (**B**) Phylogeny of the MamE clade of HtrA proteases. Numbers represent the posterior probability determined by PhyloBayes. Circles represent the degree of support from 300 bootstrap replicates in RAxML. Black: >90% support; white: >80% support. MamE sequences are colored based on the class of the associated organism, and the catalytic triad residues are shown in parentheses after each name.

While the finding that MamO has lost its protease activity to become a metal binding protein in AMB-1 was quite intriguing, we wanted to know whether this mechanism was conserved in other organisms. Due to the fact that magnetotactic *Nitrospirae* and *Omnitrophica* have not been isolated in culture, we focused our analysis on the *Proteobacteria* for which numerous representatives are available in pure-culture. Examining available whole-genome sequences indicated that all magnetotactic strains from the α-, δ- and γ-*Proteobacteria* contain two predicted trypsin-like proteases in their MAIs. We attempted to understand the evolutionary history of these proteins by including them in a large phylogenetic tree of the bacterial trypsin-2 superfamily (Methods). Within this tree, the MamO sequences from each magnetotactic α-*Proteobacterium* form a distinct, monophyletic clade (Fig 7A). Each protein has a degenerate catalytic triad, a nonglycine residue at position 193, and a bulky tryptophan in the L1 loop. The metal binding positions in LC and helix 2 also appear conserved. Although one strain has an aspartate at position 263, aspartates are also common metal coordinating residues, and we predict that this H-D motif can also participate in metal binding. We conclude that the MamO family evolved specifically in α-*Proteobacterial* magnetotactic organisms to be metal-binding pseudoproteases (S8 Fig).

In addition to the MamO proteins, we identified a second magnetosome-specific clade in the trypsin tree (Fig 7A). We named this group the MamE clade because it contains the MamE sequence from the MAI of each representative of the α-*Proteobacteria*. This group also features two predicted trypsin-like proteases from the MAI of each species of the δ- and γ-*Proteobacteria*, renamed MamE1 and MamE2 here (S3 Table). The sequence phylogeny indicates that the δ- and γ-*Proteobacteria* both experienced recent duplications of their MamEs independently. In fact, there appears to have been two rounds of duplication in the δ- family. Strikingly, each duplication event has led to a degenerate catalytic triad in one of the sequences, showing that duplication and loss of function occurred three separate times in the MamE family (Figs 7B and S9). While these inactive MamEs do not have the dihistidine motif identified in MamO, we cannot rule out the possibility that they bind metals by another mechanism. Regardless, our results imply the existence of selective pressure for pairing active and inactive proteases as each major clade of magnetotactic bacteria has evolved this feature independently.

## Discussion

Magnetotactic bacteria control the growth of their associated magnetite crystals with a level of precision that cannot be replicated in vitro. The molecular details of how they perform this task can reveal novel bioinorganic interfaces and be exploited for improved synthesis of nanomaterials. Genetic analysis has shown that magnetite biomineralization is surprisingly complex. It requires over 15 factors in AMB-1, nearly all of which are predicted integral membrane proteins [2,19,34]. A subset of these is required for the initial crystallization of iron within the magnetosome compartment [10]. While the key players for this step are known, their biochemical functions have only been inferred from sequence homology, leaving the mechanism of how magnetite biosynthesis begins a mystery.

Here, we examined two magnetite nucleation factors: the putative HtrA proteases MamE and MamO. We find that the presence of both MamE and MamO is required for the proteolysis of three biomineralization factors, MamE, MamO, and MamP. These events depend on an intact catalytic triad from MamE but not MamO, indicating that MamO activates MamE in a noncatalytic manner. Thus far, we have not been able to detect a physical interaction between MamE and MamO, but we have found that the C-terminal TauE-like transporter domain of MamO is required for activation. The putative ion transport activity of this domain could be the feature that promotes proteolysis. This model is attractive because it does not require a direct interaction between the two proteins. Indirect evidence suggests that TauE family proteins transport sulfite or sulfur containing organic ions, leading us to speculate that the concentrations of specific solutes in the magnetosome might control MamE’s activity [35,36].

Separately, we discovered that a metal-binding function in the protease domain of MamO is required for the initiation of magnetite biomineralization. In our structure, H148 and H263 directly coordinate a single metal ion. Disrupting these residues alters the binding behavior in a modified tmFRET assay, but the effect is unusual in that it lowers the FRET efficiency while slightly increasing the overall affinity for metal. Using the Förster equation and reported radius for Ni^2+^, we calculated that the fluorophore to metal distance changes from 12.8 Å in wild-type to 15.2 Å in the H148A/H263A mutant, suggesting that the binding geometry changes in a way that allows metal binding in the same vicinity [37]. We favor the explanation that the dihistidine motif identified here is part of a more complex metal coordinating network. Our soaking strategy may have missed additional sites that are inaccessible in our crystal form, and separate attempts at characterizing a fully metal-bound state using co-crystallization have been unsuccessful. Nevertheless, we identified dihistidine motif using the structure that contributes to an unexpected transition metal-binding activity in MamO.

Despite the presence of other binding features, metal binding through H148 and H263 is absolutely required in vivo as disruption of either residue completely abolishes magnetite formation. Our structural analysis shows that MamO has lost its ability to perform proteolysis altogether, supporting the idea that metal binding is now the central function of the protease domain. Consistent with this, T225, the predicted catalytic nucleophile, is completely dispensable for biomineralization. Though disrupting H116 and D149 in the predicted catalytic triad causes conditional crystal maturation defects, magnetite nucleation is not affected. Interestingly, H116 and D149 participate in a hydrogen bond on the opposite face of loop LC from the H148/H263 metal binding motif, suggesting that the conditional phenotypes could be due to temperature-dependent flexibility near the metal binding site (S5 Fig). A potential link between the two motifs is consistent with the reported inhibition of protease activity through a highly analogous metal binding site in the kallikrein family that rearranges the H-D catalytic pair (S5 Fig).

**Fig 8:**
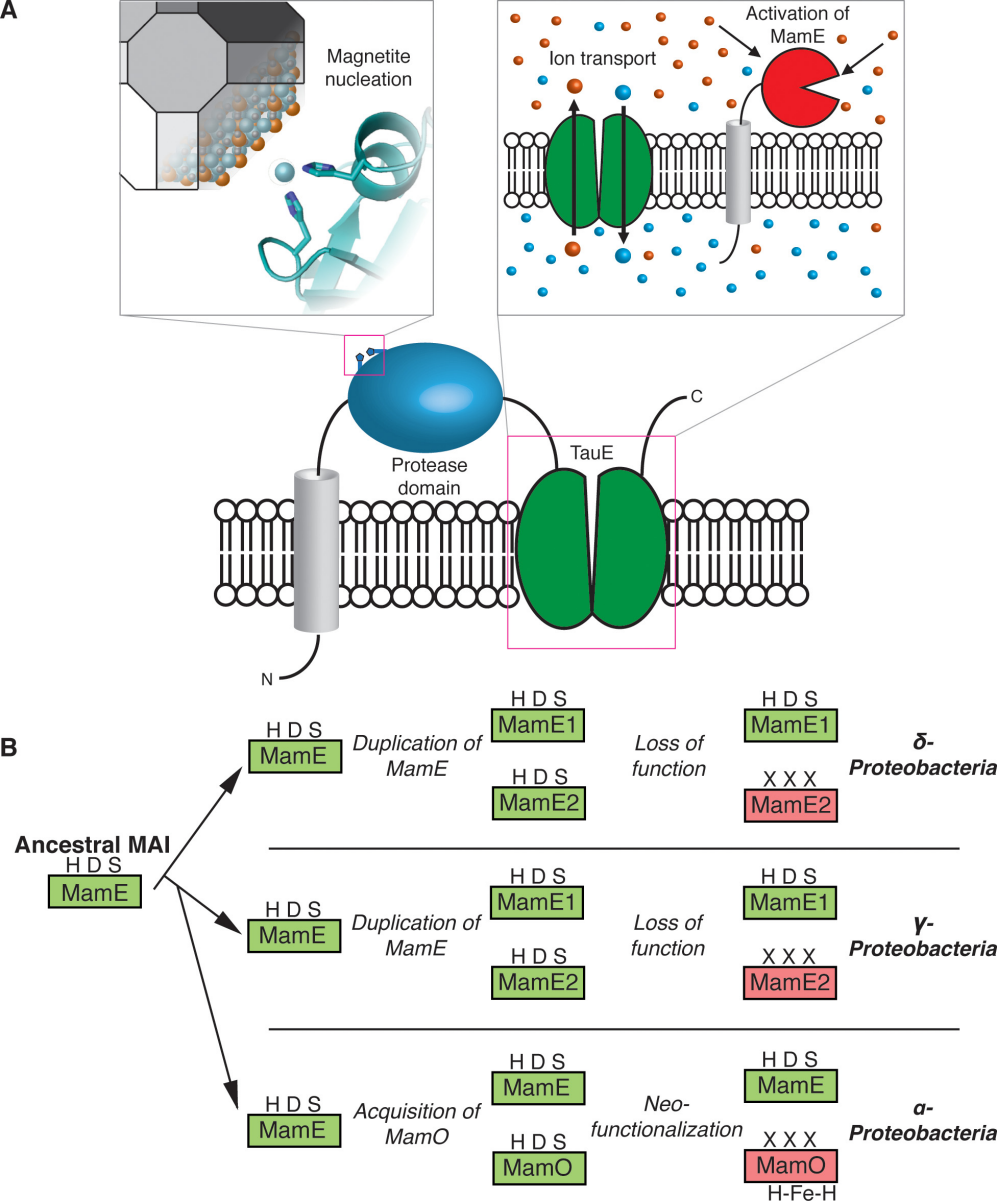
MamO in magnetosome formation and evolution. (A) A dual role in biomineralization. Distinct regions of the protein contribute to each activity separately. The protease domain promotes nucleation by binding iron and the TauE domain manipulates solute conditions that regulate MamE’s activity. (B) Specialization of the trypsin-like protease family in magnetotactic bacteria through gene duplication and subsequent neofunctionalization.

While templating of magnetite growth via an interaction between biomineralization factors and the mineral surface has been proposed, our findings with MamO emphasize that direct interactions with individual solute ions also play a role [34,38,39]. One of the most fascinating aspects of MamO’s metal ion interaction is that the H148A and H263A forms of MamO maintain the ability to bind metals but cannot support any magnetite biosynthesis in vivo. It appears that binding is insufficient and that the precise coordination geometry must be maintained, leading us to speculate that MamO directly promotes nucleation by guiding individual iron atoms into the magnetite lattice. This model is consistent with the phenotypes observed in vivo, the modest binding affinity and the surface exposed nature of the simple dihistidine motif. Additionally, it agrees with topological predictions for MamO placing the protease domain in periplasm, which is continuous with the magnetosome lumen in AMB-1 [18]. More broadly, our results define an unexpected mechanism for MamO in biomineralization. It appears to have lost the ability to perform serine protease activity and instead performs two noncatalytic functions: direct metal binding to promote magnetite nucleation and activation of MamE’s proteolytic activity (Fig 8A).

In addition to the surprising mechanism for MamO in AMB-1, we uncovered a fascinating evolutionary expansion of the trypsin family within magnetotactic bacteria. Our analysis suggests that the ancestral MAI contained a single trypsin-like protease homologous to MamE. The δ- and γ-*Proteobacteria* experienced independent duplications of this ancestral enzyme, while the α-*Proteobacteria* appear to have acquired a second, distantly related trypsin-like protease. Despite these different origins, having two redundant proteases seems to have allowed one copy to lose its catalytic ability in all three clades (Fig 8B). In α-*Proteobacteria*, MamO specialized to promote biomineralization through the two noncatalytic activities identified here. While the pseudoproteases in the other clades remain uninvestigated, the fact that inactive copies are retained strongly suggests that they also play important noncatalytic roles. The pathway that led to convergent evolution of pseudoproteases in magnetotactic organisms highlights the critical role duplication and redundancy play in facilitating diversification of protein function [40].

Perhaps more intriguing is the fact that MamO’s metal binding motif is placed at the same site on the chymotrypsin fold as the highly analogous zinc-binding site seen in the distantly related kallikreins [31,32]. This hints toward the possibility that the ability to bind metals may be a latent biochemical function carried within the fold. Such activities are absent in specific evolutionary states of a protein but can quickly surface under selective pressure [41,42]. Consistent with this, trypsin-like proteases utilize catalytic residues on loops that are well separated from the core, a property termed fold polarity that correlates with the capacity for functional diversification [43,44]. Perhaps neofunctionalization of the trypsin scaffold within magnetotactic organisms is due to an inherent stability and adaptability in the fold that makes it a useful building block for biochemical innovation.

## Methods

### Strains, Plasmids, and Culture Conditions

The strains and plasmids used in this study are listed in S4 and S5 Tables, respectively. For general maintenance and genetic manipulation, *M*. *magneticum* AMB-1 was grown in MG medium supplemented with ferric malate (30 μM). 0.7% agar was used in plates, and kanamycin was used for antibiotic selection at a concentration of 7 μg/mL (solid) or 10 μg/mL (liquid). For sucrose counterselection, MG plates contained 2% sucrose. Cultures for magnetic response measurements, western blotting, or TEM were grown in 10 mL MG medium containing 25 mM HEPES buffer (pH 7.2) and ferric malate under a 10% oxygen atmosphere. For comparing the temperature dependence of magnetic response, the strains were treated as above, except that they were grown under anaerobic atmosphere. The magnetic response of each culture was assessed using the Coefficient of Magnetism (C_mag_), which was measured as described [18].

### Genetic Manipulation

For complementation of deletion mutants, we used a modified form of pAK253 [10]. This plasmid contains a neutral region of the AMB-1 genome and integrates as a single copy at this site. Each allele is inserted under the control of the *mamAB* promoter, allowing the constitutive expression of each protein. To create the Δ*mamE*Δ*mamO*Δ*R9* (Δ*E*Δ*O*) strain, plasmid pAK243 (*sacB*-based counterselection system for deleting *mamE*) was transformed into strain AK94 (Δ*mamO*Δ*R9*), and initial integrations were selected using kanamycin. The resulting strains were grown to stationary phase in MG medium without antibiotic selection and plated on sucrose for counterselection. Deletion of *mamE* was confirmed by antibiotic sensitivity, PCR analysis, and complementation of the magnetic phenotype upon reintroduction of both *mamE* and *mamO*.

### Protein Analysis

Cultures of AMB-1 were grown to late-log phase and harvested by centrifugation at 6k x *g*. The resulting pellets were resuspended in PBS. The cell suspensions were mixed with an equal volume of 4x SDS Loading buffer and heated for 10 min at 75°C. The lysates were separated on SDS-PAGE and transferred to PVDF. The membranes were blotted and visualized using standard western blotting techniques. Polyclonal antibodies to MamE and MamP were raised in rabbits against recombinant forms of the soluble portion of each protein [25]. The monoclonal anti-FLAG antibody was purchased from Sigma.

### Transmission Electron Microscopy (TEM)

For TEM, cultures were grown to late-log phase. 1 mL from each culture was pelleted at 16k x *g*, and the pellet was resuspended in the residual medium. Cell suspensions were spotted on formvar-coated copper grids, rinsed, dabbed dry, and stored at room temperature until imaging. Imaging was performed with a FEI Tecnai 12 TEM at an accelerating voltage of 120 kV. For each strain, 15–20 cells totaling >200 crystals were analyzed.

### Protein Expression

The protease domain of *Mm*MamO (residues 45–261) was cloned into mcsII of pETDuet to create pAK876 for expression without a tag. BL21 Codon Plus cells transformed with pAK876 were grown at 37°C in 2xYT with carbenicillin (100 μg/mL) and chloramphenicol (25 μg/mL) until the OD_600_ reached 1.0. The cultures were then equilibrated at 20°C for 30 min, induced with 0.125 mM IPTG, and grown overnight. Cells were harvested by centrifugation, resuspended in Buffer A (25 mM Tris-HCl pH7.4, 400 mM NaCl, 5 mM Imidazole, 10% glycerol) supplemented with 1 μg/mL pepstatin A, 1 μg/mL leupeptin, and 0.5 mM DTT, frozen in liquid nitrogen, and stored at -80°C.

### Protein Purification

For crystallography, the frozen cell suspension was thawed on ice, lysed by sonication, and clarified at 13,000 x *g* for 30 min. The supernatant was loaded on a 3 mL Ni-NTA column, which was then washed with five column volumes of Buffer B (25 mM Tris-HCl pH 7.4, 500 mM NaCl, 10 mM Imidazole, 10% glycerol). The protein binds to Ni-NTA with its native metal binding site. After elution with one column volume Buffer C (25 mM Tris-HCl pH 7.4, 250 mM NaCl, 250 mM Imidazole, 10% glycerol), the sample was dialyzed overnight against Buffer D (25 mM Tris-HCl pH 8.0, 50 mM NaCl, 10% glycerol, 0.5 mM EDTA) and loaded onto a 1 mL HiTrap CaptoQImpRes column. The column was developed to Buffer E (25 mM Tris-HCl pH 8.0, 1 M NaCl, 10% glycerol), and the peak fractions containing the MamO protease domain were pooled, exchanged into Storage Buffer (25 mM Tris-HCl pH 7.4, 300 mM NaCl, 10% glycerol), concentrated to approximately 20 mg/mL and frozen in liquid nitrogen for storage at -80°C.

For tmFRET, the expression was performed as above except that the various mutants of the *Mm*MamO protease domains were expressed as fusions to a C-terminal strepII tag. The lysate was prepared as above and loaded onto a 1 mL StrepTrap HP column, which was washed with five column volumes of Buffer F (25 mM Tris-HCl pH 7.4, 250 mM NaCl, 10% glycerol) and eluted with three column volumes of Buffer F supplemented with 2.5 mM desthiobiotin. The eluate was concentrated and loaded onto a 16/60 Superdex 200 column and developed in Storage Buffer. The peak fractions were pooled, concentrated, and used immediately for fluorescent labeling. The purity of all proteins was confirmed by SDS-PAGE.

### Crystallization and Structure Determination

Frozen aliquots of untagged MamO protease domain were thawed on ice and exchanged to Buffer G (20 mM Tris-HCl pH 7.4, 150 mM NaCl, 5% glycerol, 0.5 mM DTT) while adjusting the protein concentration to 5 mg/mL in a 10 kDa cutoff Amicon ultrafilter. Crystals grew in the hanging drop vapor diffusion format after mixing the protein with an equal volume of well solution (50 mM Na-Acetate pH 4.6, 3.6 M NH_4_Cl, 5% glycerol) and equilibrating against 1 mL well solution at 18°C. Cubic crystals of MamO appeared in 1–2 d and grew to their full size after 4–6 wk. Each crystal was cryoprotected with a solution of 50 mM Na-Acetate pH 4.6, 3.6 M NH_4_Cl, and 22% glycerol before plunge-freezing in liquid N_2_.

For nickel soaking, NiCl_2_ was added directly to drops containing fully-grown crystals for a 10 mM final concentration. The wells were resealed and the crystals soaked overnight. At harvest, each crystal was passed through three 1 min soaks in a buffer containing 50 mM Tris-HCl pH 8.0, 3.6 M NH_4_Cl, 22% glycerol, and 10 mM NiCl_2_ before plunge-freezing.

Diffraction data was collected on beamline 8.3.1 at the Advance Light Source (Lawrence Berkeley National Laboratory, Berkeley, California). Indexing and scaling was performed using HKL2000 [45]. The apo- structure was solved by molecular replacement in Phaser with the protease domain of *Ec*DegP (1KY9) as a search model [46]. The Ni-bound structure was solved using the apo-MamO structure as a molecular replacement search model. Model building and refinement were carried out with alternating cycles of COOT [47] and phenix.refine [48]. Placement of the Ni ion was confirmed with an anomalous map from SAD data collected at the Ni absorption edge.

### Fluorescein-5-Maleimide (F-5-M) Labeling

For fluorescent labeling, variants of the MamO protease domain were diluted to 50 μM in 1 mL of Buffer H (50 mM NaPhosphate pH 7.2, 150 mM NaCl, 10% glycerol). Fluorescein-5-maleimide (dissolved at 50 mM in DMSO) was added to a final concentration of 1 mM, and the reaction was incubated overnight at 4°C. The reaction was quenched with DTT, exchanged into Buffer D using a PD-10 column, and loaded on a 1 mL HiTrap Q FF. The column was developed to Buffer E, and each protein eluted as a single fluorescent peak. The peak fractions were pooled, treated with 1 mM EDTA to remove any trace metal, exchanged extensively into Chelex-treated Storage Buffer, and frozen in small aliquots. Labeling efficiency was measured based on A_492_/A_280_ and was between 50% and 55% for all preparations.

### tmFRET

For metal binding experiments, all buffers were prepared in acid-washed glassware and treated with chelex resin. Each protein was diluted to 60–80 nM in fluorescence buffer (10 mM Tris-HCl pH 8.0, 150 mM NaCl). Metal solutions were prepared at 10X concentration in chelex-treated H_2_O. Protein was dispensed into 96-well plates, and the plates were scanned for fluorescence emission in a Tecan Infinite 200 plate reader in top-read mode (Ex: 492 nm; Em: 505–570 nm). Metal solutions were then diluted into each well at the appropriate concentration and the plate was rescanned. Due to the spontaneous oxidation of ferrous iron in ambient atmosphere, all iron binding experiments were performed in the absence of oxygen. For iron binding, all solutions were prepared using anoxic liquids. The samples were prepared under anoxic atmosphere in a clear-bottom 96-well plate. To prevent introduction of oxygen, the wells were sealed by covering the plate with a black adhesive cover. The plate was then removed from the anaerobic chamber and scanned using bottom-read mode.

Each measurement was performed on independently prepared solutions in quadruplicate. The metal-quenched fluorescence spectrum from each well was normalized to the fluorescence before metal addition. F_metal_/F represents the normalized fluorescence averaged from an 11 nm window around the peak. After plotting F_metal_/F as a function of metal concentration, each curve was fit to a two-site model (below), in which K_d2_ and E represent the dissociation constant and FRET efficiency for metal binding by MamO, respectively. K_d1_ represents a nonspecific, solution-based quenching component [37].

$$\frac{F_{metal}}{F}=\left(1-\frac{E}{1+\frac{{Kd}_{2}}{\left[metal\right]}}\right)\left(\frac{1}{1+\frac{\left[metal\right]}{{Kd}_{1}}}\right)$$

### Ni-NTA Binding Assays

StrepII-tagged forms of the MamO protease domain were purified as described for the tmFRET experiments with the omission of the F-5-M labeling and ion exchange steps. Proteins were diluted to approximately 15 μM in Column Buffer (25 mM Tris-HCl pH 7.4, 300 mM NaCl, 5 mM Imidazole, 10% glycerol) and loaded onto a 0.5 mL Ni-NTA column equilibrated in column buffer. The column was washed with ten column volumes of Column Buffer and eluted with two column volumes Buffer C. Binding was assessed by separating the fractions on 12% SDS-PAGE and staining with Coomassie Blue.

### Phylogenetic Analysis

To understand the phylogeny of Mam proteases, we took a broad approach, characterizing their location within the trypsin-like protease family. The *Trypsin_2* Pfam (PF13365) was used to generate a hidden Markov model (HMM) that was used to search a database composed of protein sequences from approximately 2,100 bacterial and archaeal genomes along with the Mam proteases [49]. This search was performed using hmmscan (HMMER3.1b1, hmmer.janelia.org), retaining all hits with an e-value for the entire sequence less than 10^−5^, identifying 6,104 proteins. The hits were clustered using CD-HIT with a sequence similarity cutoff of 0.8, yielding 3,431 sequences [50]. These were aligned using MUSCLE (v3.8.31) with the maxiters parameter set to 2 [51]. The resulting alignment was trimmed using Gblocks, using parameters appropriate for divergent datasets as described by Sassera et al. [52,53]. This alignment was then used to generate a phylogeny using FastTree 2 (v2.1.7) with the default settings [54]. However, this alignment contained only six informative positions. To improve the quality of the alignment, we iteratively removed long branches from the tree and regenerated the alignment. After removing 90 taxa over four iterations, we settled on an alignment with 18 conserved positions.

Two clear branches emerged from this analysis. One branch ('the Deg branch') contained DegP, DegQ, and DegS sequences from the γ-*Proteobacteria*, in addition to most of the MamE sequences. The bottom branch contained the YdgD sequence from *Escherichia coli*. We extracted the sequences from the Deg branch (843 sequences) and generated an alignment (46 positions) and tree using the methods described above (Fig 7A). In this tree, the MamE sequences appeared to be closely related, but their phylogenetic relationships were ambiguous. Additionally, we realized that a subset of δ-proteobacterial trypsin-like sequences were falsely excluded from the MamE branch due to substitutions in the catalytic triad that obscured the phylogenetic signal in the short alignment. Additional phylogenetic testing confirmed the relationship of these four sequences to the canonical MamE sequences, so they were merged into the MamE branch.

We extracted the MamE sequences and aligned them using five DegS sequences from the γ-*Proteobacteria*, which appeared to be closely related based on the phylogeny in Fig 7B. This alignment was much larger (172 positions), and the resulting tree from FastTree 2 gave well-supported interior nodes for the MamE branch. We used two other phylogenetic methods to test this phylogeny. First, we used ProtTest 3.0 to select the best substitution matrix (in this case, the WAG model) and performed 100 independent inferences with 300 bootstraps in RAxML [55,56]. Secondly, we used the nonparametric Monte Carlo Markov Chain algorithm PhyloBayes 3 to generate a tree not based on prior assumptions about the site-specific evolution of the MamE sequences [57]. A summary tree integrating the results from PhyloBayes and RAxML is depicted in Fig 7B. Additionally, we rooted the MamE branch to five closely related sequences from the Clostridiales, and this, too, strongly supported the phylogeny in Fig 7B according to both PhyloBayes and RAxML.

## Supporting Information

S1 Data Biomineralization data for Figs 2, 6, S1, S2 and S3. (XLSX)

S2 Data tmFRET data for Figs 6 and S7. (XLSX)

S1 Fig Temperature dependence of magnetic response for mamO alleles in this study. (TIF)

S2 Fig Characterization of strains from Fig 3. Stars indicate N-terminally 3xFLAG-tagged alleles throughout Fig 3. In the histograms, each measurement represents the average of three biological replicates. Error bars represent the standard deviation of the replicates. (**A**) Processing of MamO alleles used in this study in the Δ*O*Δ*R9* background. (**B**) Magnetic response of the strains in A. (**C**) Proteolytic processing of MamO requires the MamE active site. (**D**) Magnetic response of the strains from C.
(TIF)

S3 Fig Cross-complementation of MamO protease domain mutants by LimO. (**A**) *limO* is contained within a partially duplicated region of the *mamAB* cluster termed R9. While *mamO* is predicted to have a trypsin like-protease domain and a TauE-like transporter domain, *limO* has only a predicted trypsin-like domain with 98% identity to the N-terminus of *mamO*. *LimO* contains all of the critical residues identified in MamO in this study. (**B**) MamO alleles are proteolytically processed identically in the single Δ*mamO* strain as they are in the Δ*O*Δ*R9* background. (**C**) All of the *mamO* alleles examined in this work restore wild-type biomineralization in the single Δ*mamO* background, showing that *limO* encodes a fully functional copy of the *mamO* protease domain.
(TIF)

S4 Fig Structure of the MamO protease domain. (**A**) Schematic of the chymotrypsin fold with the loops and catalytic residues indicated. (**B**) Comparison of the L1 loop in MamO to the trypsin family consensus. (**C**) Overall structure of the MamO protease domain solved to 2.6 Å. The catalytic residues and bound peptides are show in stick representation.
(TIF)

S5 Fig Similarity of the MamO metal binding site to equine kallikrein-3. (**A**) *F*_*O*_ *-F*_*C*_ omit map showing the bound Ni^2+^ ion in MamO. Blue: Ni^2+^; red: H_2_O; green: Cl^-^. (**B,C**) Comparison of metal binding sites in MamO and equine kallikrein-3. Coordinates of the zinc-bound structure reported in Carvahlo et al. [31] were not deposited in the PDB.
(TIF)

S6 Fig Fluorescein-5-maleimide labeling of MamO^Q258C^. (**A**) The fluorescent labeling site in MamO was chosen based on the optimum FRET distance from Taraska et al. [33] (**B**) Purification and fluorescent labeling of MamO^Q258C^ and MamO^Q258C H148A H263A^.
(TIF)

S7 Fig tmFRET analysis of metal binding. (**A**) Fluorescence quenching of MamO Q258C labeled with fluorescein-5-maleimide in the presence of increasing concentrations of NiSO_4_. (**B**) Binding of various transition metals to labeled MamO. Error bars represent the standard deviation from four independent measurements. The dotted lines are fits to the binding equation described in Methods. (**C**) Binding constants from tmFRET experiments. (**D**) Ni-NTA affinity assays with purified protease domains.
(TIF)

S8 Fig Alignment of the MamO family. The conservation of critical residues discussed in the text is indicated with colored boxes.
(TIF)

S9 Fig Sample analysis of representative trypsin-like sequences from magnetotactic bacteria. (**A**) Alignment of the catalytic loops from a set of trypsin-like sequences. The trypsin sequences from four magnetotactic organisms were aligned with two canonical HtrAs, *H*. *sapiens* HtrA1 and *E*. *coli* DegP. Positions of catalytic triad residues are marked with a star. (**B**) Phylogeny of the sequences inferred from the detailed analysis shown in Fig 7. The identities of the catalytic triad residues are shown in parentheses after each protein name. Boxes represent the class level taxonomy of the organism within the *Proteobacteria*.
(TIF)

S1 Table Summary of crystallographic data. (DOCX)

S2 Table L1 loop conformation in a set of trypsin-like protease structures. The main chain configuration at position 193 for all chains in the asymmetric unit of each structure was used to generate the table.
(DOCX)

S3 Table Suggested renaming of the MamE clade. The naming in a previous comparative genomic study is unconventional due to confusion over analogy to the MAI in α-*Proteobacteria* [12]. Our phylogenetic analysis clarifies the ancestry and allows us to use accepted nomenclature.
(DOCX)

S4 Table Strains used in this study. (DOCX)

S5 Table Plasmids used in this study. (DOCX)

## Abbreviations

MAI: magnetosome island
SAD: single wavelength anomalous dispersion
TEM: transmission electron microscopy
tmFRET: transition metal ion Förster Resonance Energy Transfer

## References

[1] Weiner S. Biomineralization: A structural perspective. Journal of Structural Biology. 2008;163: 229–234. 10.1016/j.jsb.2008.02.001 18359639

[2] Komeili A. Molecular mechanisms of compartmentalization and biomineralization in magnetotactic bacteria. FEMS Microbiology Reviews. 2011;36: 232–255. 10.1111/j.1574-6976.2011.00315.x PMC3540109 22092030

[3] Blakemore R. Magnetotactic Bacteria. Science. 1975;190: 377–379. 170679 10.1126/science.170679

[4] Balkwill DL, Maratea D, Blakemore RP. Ultrastructure of a magnetotactic spirillum. J Bacteriol. American Society for Microbiology (ASM); 1980;141: 1399–1408. 10.1016/j.cell.2011.02.024 PMC293838 6245069

[5] Lefèvre CT, Bennet M, Landau L, Vach P, Pignol D, Bazylinski DA, et al Diversity of Magneto-Aerotactic Behaviors and Oxygen Sensing Mechanisms in Cultured Magnetotactic Bacteria. Biophysj. The Authors; 2014;107: 527–538. 10.1016/j.bpj.2014.05.043 PMC4104051 25028894

[6] Lefevre CT, Bazylinski DA. Ecology, Diversity, and Evolution of Magnetotactic Bacteria. Microbiology and Molecular Biology Reviews. 2013;77: 497–526. 10.1128/MMBR.00021-13 24006473 PMC3811606

[7] Fukuda Y, Okamura Y, Takeyama H, Matsunaga T. Dynamic analysis of a genomic island in Magnetospirillum sp. strain AMB-1 reveals how magnetosome synthesis developed. FEBS Letters. 2006;580: 801–812. 10.1016/j.febslet.2006.01.003 16423350

[8] Schubbe S, Williams TJ, Xie G, Kiss HE, Brettin TS, Martinez D, et al Complete Genome Sequence of the Chemolithoautotrophic Marine Magnetotactic Coccus Strain MC-1. Applied and Environmental Microbiology. 2009;75: 4835–4852. 10.1128/AEM.02874-08 19465526 PMC2708431

[9] Schubbe S, Kube M, Scheffel A, Wawer C, Heyen U, Meyerdierks A, et al Characterization of a Spontaneous Nonmagnetic Mutant of Magnetospirillum gryphiswaldense Reveals a Large Deletion Comprising a Putative Magnetosome Island. J Bacteriol. 2003;185: 5779–5790. 10.1128/JB.185.19.5779–5790.2003 13129949 PMC193972

[10] Murat D, Quinlan A, Vali H, Komeili A. Comprehensive genetic dissection of the magnetosome gene island reveals the step-wise assembly of a prokaryotic organelle. Proc Natl Acad Sci U S A. 2010;107: 5593–5598. 10.1073/pnas.0914439107 20212111 PMC2851823

[11] Lohße A, Ullrich S, Katzmann E, Borg S, Wanner G, Richter M, et al Functional Analysis of the Magnetosome Island in Magnetospirillum gryphiswaldense: The mamAB Operon Is Sufficient for Magnetite Biomineralization. Battista JR, editor. PLoS ONE. 2011;6: e25561–10. 10.1371/journal.pone.0025561 22043287 PMC3197154

[12] Lefèvre CT, Trubitsyn D, Abreu F, Kolinko S, Jogler C, de Almeida LGP, et al Comparative genomic analysis of magnetotactic bacteria from the Deltaproteobacteriaprovides new insights into magnetite and greigite magnetosome genes required for magnetotaxis. Environ Microbiol. 2013;15: 2712–2735. 10.1111/1462-2920.12128 23607663

[13] Lefèvre CT, Trubitsyn D, Abreu F, Kolinko S, de Almeida LGP, de Vasconcelos ATR, et al Monophyletic origin of magnetotaxis and the first magnetosomes. Environ Microbiol. 2013;15: 2267–2274. 10.1111/1462-2920.12097 23438345

[14] Grunberg K, Muller EC, Otto A, Reszka R, Linder D, Kube M, et al Biochemical and Proteomic Analysis of the Magnetosome Membrane in Magnetospirillum gryphiswaldense. Applied and Environmental Microbiology. 2004;70: 1040–1050. 10.1128/AEM.70.2.1040–1050.2004 14766587 PMC348919

[15] Zarivach R. Structure prediction of magnetosome-associated proteins. Front Microbiol. 2014: 1–17. 10.3389/fmicb.2014.00009/abstract PMC3905215 24523717

[16] Rahn-Lee L, Komeili A. The magnetosome model: insights into the mechanisms of bacterial biomineralization. Front Microbiol. 2013: 1–8. 10.3389/fmicb.2013.00352/abstract PMC3840617 24324464

[17] Komeili A, Vali H, Beveridge TJ, Newman DK. Magnetosome vesicles are present before magnetite formation, and MamA is required for their activation. Proc Natl Acad Sci U S A. 2004;101: 3839–3844. 10.1073/pnas.0400391101 15004275 PMC374331

[18] Komeili A, Li Z, Newman DK, Jensen GJ. Magnetosomes Are Cell Membrane Invaginations Organized by the Actin-Like Protein MamK. Science. 2006;311: 242–245. 10.1126/science.1123231 16373532

[19] Arakaki A, Yamagishi A, Fukuyo A, Tanaka M, Matsunaga T. Co-ordinated functions of Mms proteins define the surface structure of cubo-octahedral magnetite crystals in magnetotactic bacteria. Mol Microbiol. 2014;93: 554–567. 10.1111/mmi.12683 24961165

[20] Lohße A, Borg S, Raschdorf O, Kolinko I, Tompa E, Posfai M, et al Genetic Dissection of the mamAB and mms6 Operons Reveals a Gene Set Essential for Magnetosome Biogenesis in Magnetospirillum gryphiswaldense. J Bacteriol. 2014;196: 2658–2669. 10.1128/JB.01716-14 24816605 PMC4097598

[21] Clausen T, Kaiser M, Huber R, Ehrmann M. HTRA proteases: regulated proteolysis in protein quality control. Nat Rev Mol Cell Biol. Nature Publishing Group; 2011;12: 152–162. 10.1038/nrm3065 21326199

[22] Yang W, Li R, Peng T, Zhang Y, Jiang W, Li Y, et al mamO and mamE genes are essential for magnetosome crystal biomineralization in Magnetospirillum gryphiswaldense MSR-1. Res Microbiol. Elsevier Masson SAS; 2010;161: 701–705. 10.1016/j.resmic.2010.07.002 20674739

[23] Quinlan A, Murat D, Vali H, Komeili A. The HtrA/DegP family protease MamE is a bifunctional protein with roles in magnetosome protein localization and magnetite biomineralization. Mol Microbiol. 2011;80: 1075–1087. 10.1111/j.1365-2958.2011.07631.x 21414040 PMC3091955

[24] Siponen MI, Legrand P, Widdrat M, Jones SR, Zhang W-J, Chang MCY, et al Structural insight into magnetochrome-mediated magnetite biomineralization. Nature. Nature Publishing Group; 2014;502: 681–684. 10.1038/nature12573 24097349

[25] Jones SR, Wilson TD, Brown ME, Rahn-Lee L, Yu Y, Fredriksen LL, et al Genetic and biochemical investigations of the role of MamP in redox control of iron biomineralization in Magnetospirillum magneticum. Proc Natl Acad Sci U S A. 2015;112: 3904–3909. 10.1073/pnas.1417614112 25775527 PMC4386411

[26] Perona JJ, Craik CS. Evolutionary Divergence of Substrate Specificity within the Chymotrypsin-like Serine Protease Fold. J Biol Chem. 1997;272: 29987–29990. 9374470 10.1074/jbc.272.48.29987

[27] Sohn J, Grant RA, Sauer RT. Allostery Is an Intrinsic Property of the Protease Domain of DegS: IMPLICATIONS FOR ENZYME FUNCTION AND EVOLUTION. J Biol Chem. 2010;285: 34039–34047. 10.1074/jbc.M110.135541 20739286 PMC2962503

[28] Wrase R, Scott H, Hilgenfeld R, Hansen G. The Legionella HtrA homologue DegQ is aself-compartmentizing protease thatforms large 12-meric assemblies. Proc Natl Acad Sci U S A. 2011;108: 10490–10495. 10.1073/pnas.1101084108/-/DCSupplemental/pnas.1101084108_SI.pdf 21670246 PMC3127897

[29] Bobofchak KM, Pineda AO, Mathews FS, Di Cera E. Energetic and Structural Consequences of Perturbing Gly-193 in the Oxyanion Hole of Serine Proteases. J Biol Chem. 2005;280: 25644–25650. 10.1074/jbc.M503499200 15890651

[30] Lovell SC, Davis IW, Arendall WB III, de Bakker PIW, Prisant MG, Richardson JS. Structure Validation by Cα Geometry: ϕ, ψ, and Cβ Deviation. Proteins. 2003;50: 437–450. 12557186 10.1002/prot.10286

[31] Carvalho AL, Sanz L, Barettino D, Romero A, Calvete JJ, Romão MJ. Crystal Structure of a Prostate Kallikrein Isolated from Stallion Seminal Plasma: A Homologue of Human PSA. J Mol Biol. 2002;322: 325–337. 10.1016/S0022-2836(02)00705-2 12217694

[32] Goettig P, Magdolen V, Brandstetter H. Natural and synthetic inhibitors of kallikrein-related peptidases (KLKs). Biochimie. Elsevier Masson SAS; 2010;92: 1546–1567. 10.1016/j.biochi.2010.06.022 PMC3014083 20615447

[33] Taraska JW, Puljung MC, Olivier NB, Flynn GE, Zagotta WN. Mapping the structure and conformational movements of proteins with transition metal ion FRET. Nature Methods. Nature Publishing Group; 2009;6: 532–537. 10.1038/nmeth.1341 PMC2738593 19525958

[34] Murat D, Falahati V, Bertinetti L, Csencsits R, Körnig A, Downing K, et al The magnetosome membrane protein, MmsF, is a major regulator of magnetite biomineralization in Magnetospirillum magneticum AMB-1. Mol Microbiol. Blackwell Publishing Ltd; 2012;85: 684–699. 10.1111/j.1365-2958.2012.08132.x PMC3570065 22716969

[35] Weinitschke S, Denger K, Cook AM, Smits THM. The DUF81 protein TauE in Cupriavidus necator H16, a sulfite exporter in the metabolism of C2 sulfonates. Microbiology. 2007;153: 3055–3060. 10.1099/mic.0.2007/009845-0 17768248

[36] Mayer J, Denger K, Hollemeyer K, Schleheck D, Cook AM. (R)-Cysteate-nitrogen assimilation by Cupriavidus necator H16 with excretion of 3-sulfolactate: a patchwork pathway. Arch Microbiol. 2012;194: 949–957. 10.1007/s00203-012-0825-y 22797525

[37] Yu X, Wu X, Bermejo GA, Brooks BR, Taraska JW. Accurate High-Throughput Structure Mapping and Prediction with Transition Metal Ion FRET. Structure. Elsevier Ltd; 2013;21: 9–19. 10.1016/j.str.2012.11.013 PMC3700372 23273426

[38] Arakaki A, Webb J, Matsunaga T. A Novel Protein Tightly Bound to Bacterial Magnetic Particles in Magnetospirillum magneticum Strain AMB-1. J Biol Chem. 2003;278: 8745–8750. 10.1074/jbc.M211729200 12496282

[39] Amemiya Y, Arakaki A, Staniland SS, Tanaka T, Matsunaga T. Controlled formation of magnetite crystal by partial oxidation of ferrous hydroxide in the presence of recombinant magnetotactic bacterial protein Mms6. Biomaterials. 2007;28: 5381–5389. 10.1016/j.biomaterials.2007.07.051 17720242

[40] Espinosa-Cantú A, Ascencio D, Barona-Gómez F, DeLuna A. Gene duplication and the evolution of moonlighting proteins. Front Genet. 2015;6: 1–7. 26217376 10.3389/fgene.2015.00227 PMC4493404

[41] Morrone D, Hillwig ML, Mead ME, Lowry L, Fulton DB, Peters RJ. Evident and latent plasticity across the rice diterpene synthase family with potential implications for the evolution of diterpenoid metabolism in the cereals. Biochem J. 2011;435: 589–595. 10.1042/BJ20101429 21323642 PMC3723722

[42] Ben-David M, Wieczorek G, Elias M, Silman I, Sussman JL, Tawfik DS. Catalytic Metal Ion Rearrangements Underline Promiscuity and Evolvability of a Metalloenzyme. J Mol Biol. Elsevier Ltd; 2013;425: 1028–1038. 10.1016/j.jmb.2013.01.009 23318950

[43] Dellus-Gur E, Toth-Petroczy A, Elias M, Tawfik DS. What Makes a Protein Fold Amenable to Functional Innovation? Fold Polarity and Stability Trade-offs. J Mol Biol. Elsevier Ltd; 2013;425: 2609–2621. 10.1016/j.jmb.2013.03.033 23542341

[44] Toth-Petroczy A, Tawfik DS. The robustness and innovability of protein folds. Current Opinion in Structural Biology. Elsevier Ltd; 2014;26: 131–138. 10.1016/j.sbi.2014.06.007 25038399

[45] Otwinoski Z, Minor W. Processing of X-Ray Diffraction Data Collected in Oscillation Mode. Meth Enzymol. 1997;276: 307–326. 10.1016/S0076-6879(97)76066-X 27754618

[46] Krojer T, Garrido-Franco M, Huber R, Ehrmann M, Clausen T. Crystal structure of DegP (HtrA) reveals a new protease-chaperone machine. Nature. 2002;416: 455–459. 11919638 10.1038/416455a

[47] Emsley P, Lohkamp B, Scott WG, Cowtan K. Features and development of Coot. Acta Crystallogr D Biol Crystallogr. International Union of Crystallography; 2010;66: 486–501. 10.1107/S0907444910007493 20383002 PMC2852313

[48] Adams PD, Afonine PV, Bunkoczi G, Chen VB, Davis IW, Echols N, et al PHENIX: a comprehensive Python-based system for macromolecular structure solution. Acta Crystallogr D Biol Crystallogr. International Union of Crystallography; 2010;66: 213–221. 10.1107/S0907444909052925 20124702 PMC2815670

[49] Eddy SR. Profile hidden Markov models. Bioinformatics. 1998;14: 755–763. 9918945 10.1093/bioinformatics/14.9.755

[50] Li W, Godzik A. Cd-hit: a fast program for clustering and comparing large sets of protein or nucleotide sequences. Bioinformatics. 2006;22: 1658–1659. 10.1093/bioinformatics/btl158 16731699

[51] Edgar RC. MUSCLE: multiple sequence alignment with high accuracy and high throughput. Nucleic Acids Research. 2004;32: 1792–1797. 10.1093/nar/gkh340 15034147 PMC390337

[52] Castresana J. Selection of Conserved Blocks from Multiple Alignments for Their Use in Phylogenetic Analysis. Mol Biol Evol. 2000;17: 540–552. 10742046 10.1093/oxfordjournals.molbev.a026334

[53] Sassera D, Lo N, Epis S, D'Auria G, Montagna M, Comandatore F, et al Phylogenomic Evidence for the Presence of a Flagellum and cbb3 Oxidase in the Free-Living Mitochondrial Ancestor. Mol Biol Evol. 2011;28: 3285–3296. 10.1093/molbev/msr159 21690562

[54] Price MN, Dehal PS, Arkin AP. FastTree 2 –Approximately Maximum-Likelihood Trees for Large Alignments. PLoS ONE. 2010;5: e9940 10.1371/journal.pone.0009490 20224823 PMC2835736

[55] Abascal F, Zardoya R, Posada D. ProtTest: selection of best-fit models of protein evolution. Bioinformatics. Oxford University Press; 2005;21: 2104–2105. 10.1093/bioinformatics/bti263 15647292

[56] Stamatakis A. RAxML Version 8: a Tool for Phylogenetic Analysis and Post-Analysis of Large Phylogenies. Bioinformatics. 2014;30: 1312–1313. 10.1093/bioinformatics/btu033/-/DC1 24451623 PMC3998144

[57] Lartillot N, Lepage T, Blanquart S. PhyloBayes 3: a Bayesian software package for phylogenetic reconstruction and molecular dating. Bioinformatics. 2009;25: 2286–2288. 10.1093/bioinformatics/btp368 19535536

