# Supplementary figures and images for: MamO Is a Repurposed Serine Protease that Promotes Magnetite Biomineralization through Direct Transition Metal Binding in Magnetotactic Bacteria
Source: PLoS Biol. 2016 Mar 16;14(3):e1002402. doi: 10.1371/journal.pbio.1002402 (PMC4794232; doi:10.1371/journal.pbio.1002402)

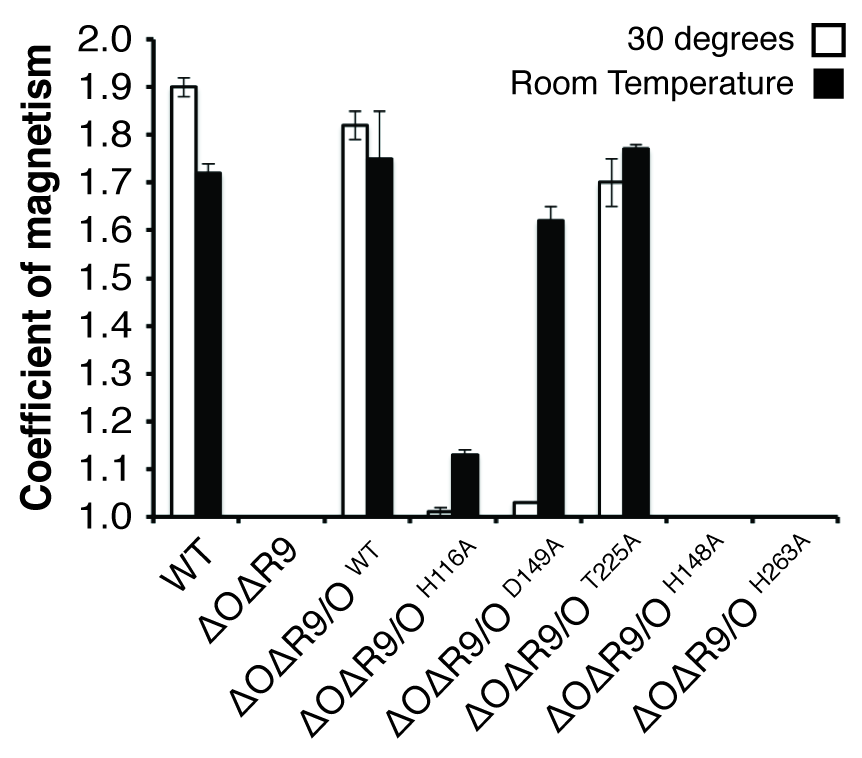

Supplement: S1 Fig — (TIF) [file pbio.1002402.s003.tif]

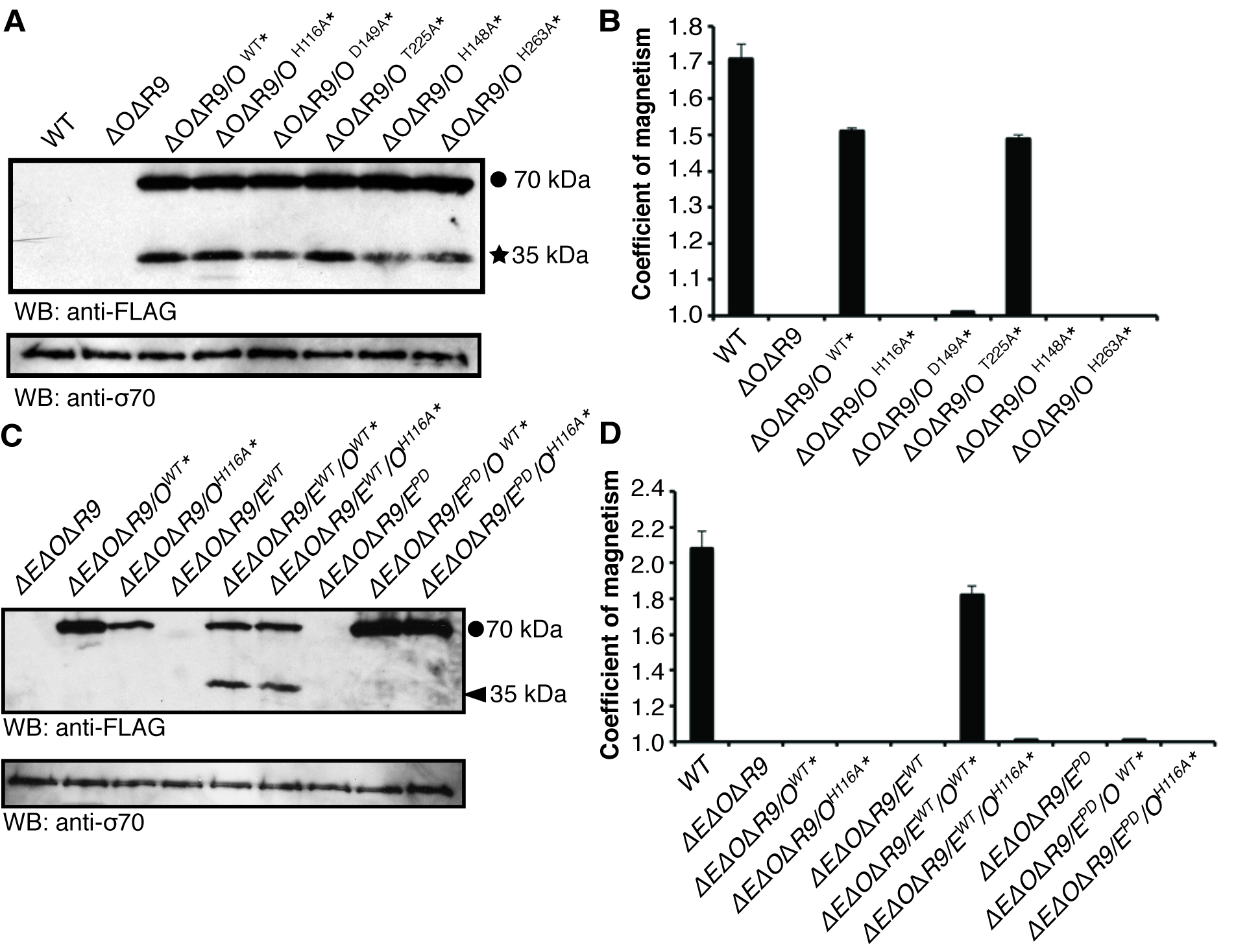

Supplement: S2 Fig — Stars indicate N-terminally 3xFLAG-tagged alleles throughout Fig 3. In the histograms, each measurement represents the average of three biological replicates. Error bars represent the standard deviation of the replicates. (A) Processing of MamO alleles used in this study in the ΔOΔR9 background. (B) Magnetic response of the strains in A. (C) Proteolytic processing of MamO requires the MamE active site. (D) Magnetic response of the strains from C. (TIF) [file pbio.1002402.s004.tif]

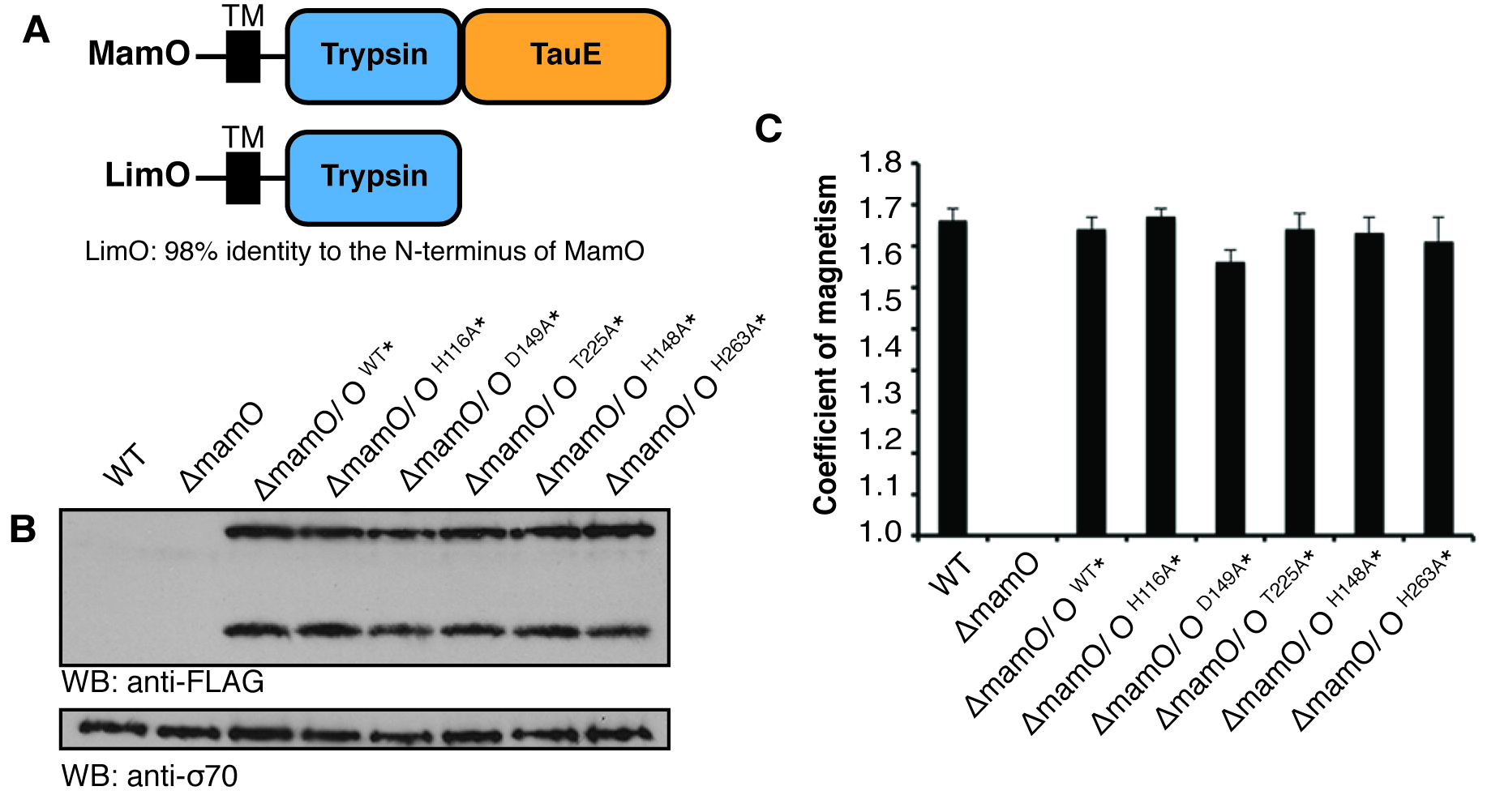

Supplement: S3 Fig — (A) limO is contained within a partially duplicated region of the mamAB cluster termed R9. While mamO is predicted to have a trypsin like-protease domain and a TauE-like transporter domain, limO has only a predicted trypsin-like domain with 98% identity to the N-terminus of mamO. LimO contains all of the critical residues identified in MamO in this study. (B) MamO alleles are proteolytically processed identically in the single ΔmamO strain as they are in the ΔOΔR9 background. (C) All of the mamO alleles examined in this work restore wild-type biomineralization in the single ΔmamO background, showing that limO encodes a fully functional copy of the mamO protease domain. (TIF) [file pbio.1002402.s005.tif]

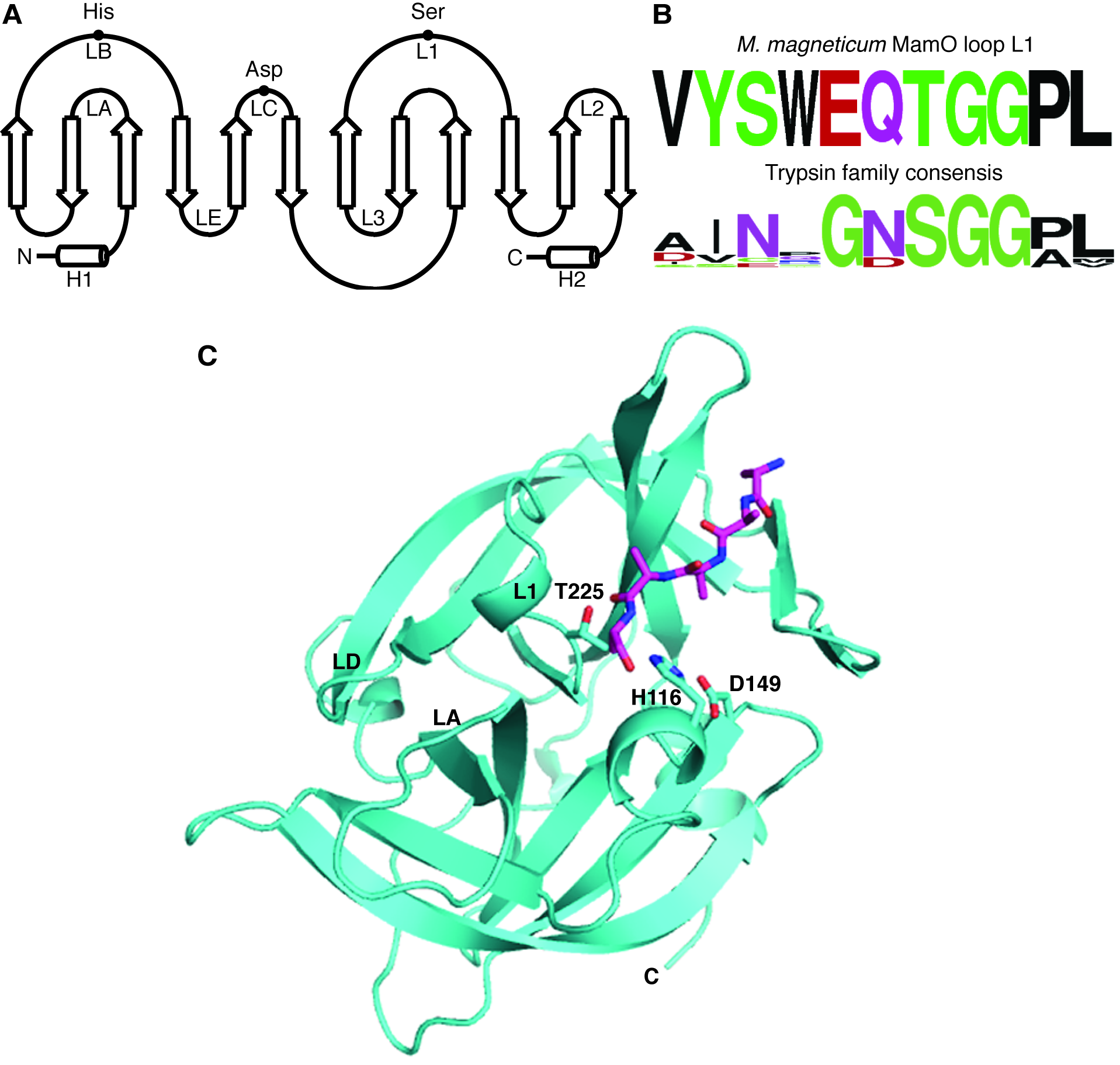

Supplement: S4 Fig — (A) Schematic of the chymotrypsin fold with the loops and catalytic residues indicated. (B) Comparison of the L1 loop in MamO to the trypsin family consensus. (C) Overall structure of the MamO protease domain solved to 2.6 Å. The catalytic residues and bound peptides are show in stick representation. (TIF) [file pbio.1002402.s006.tif]

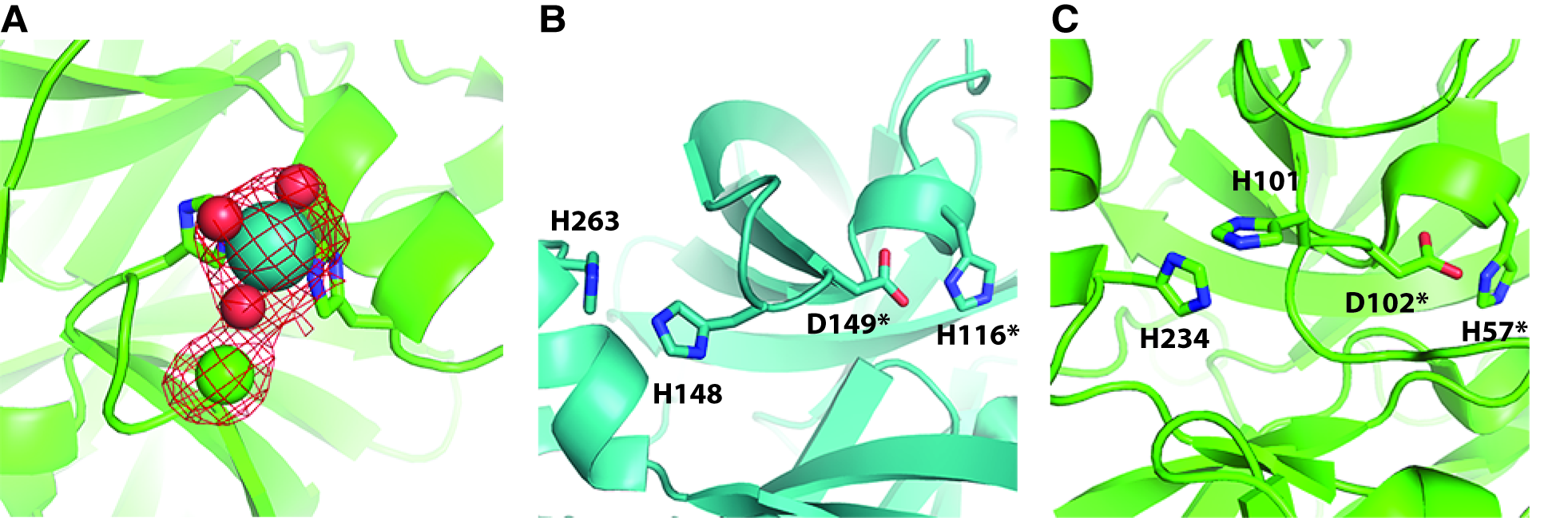

Supplement: S5 Fig — (A) F O -F C omit map showing the bound Ni2+ ion in MamO. Blue: Ni2+; red: H2O; green: Cl-. (B,C) Comparison of metal binding sites in MamO and equine kallikrein-3. Coordinates of the zinc-bound structure reported in Carvahlo et al. [31] were not deposited in the PDB. (TIF) [file pbio.1002402.s007.tif]

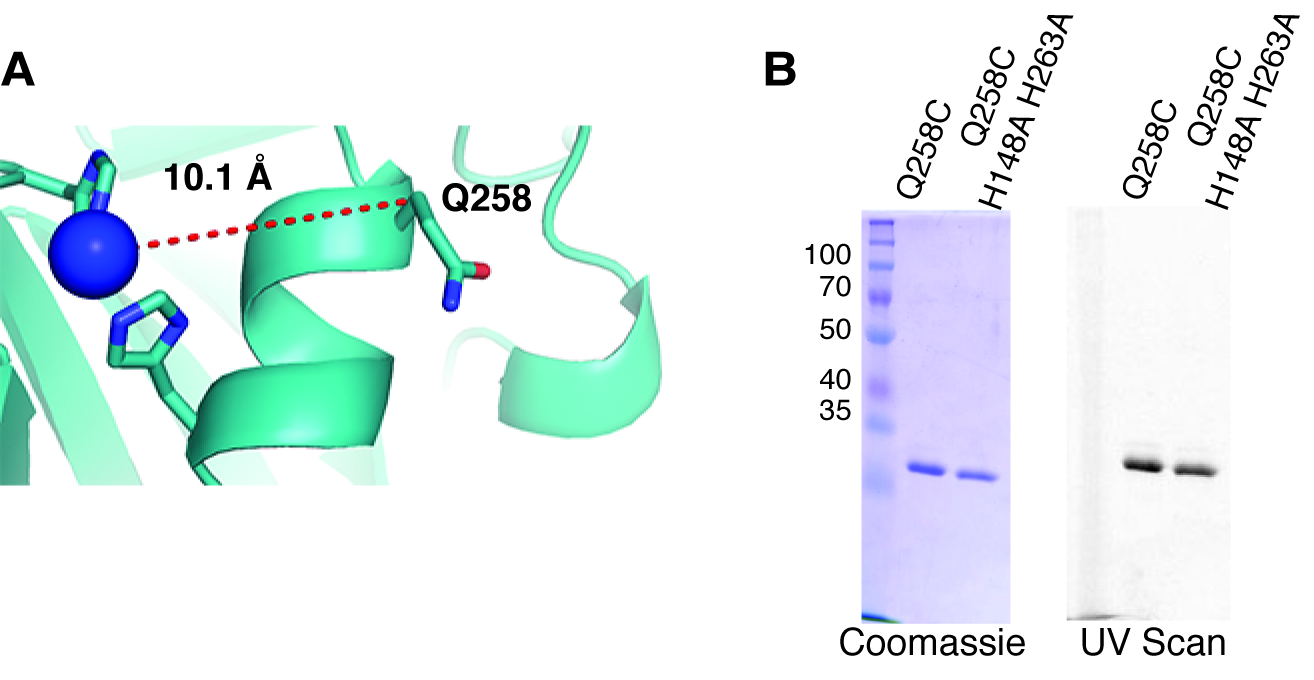

Supplement: S6 Fig — (A) The fluorescent labeling site in MamO was chosen based on the optimum FRET distance from Taraska et al. [33] (B) Purification and fluorescent labeling of MamOQ258C and MamOQ258C H148A H263A. (TIF) [file pbio.1002402.s008.tif]

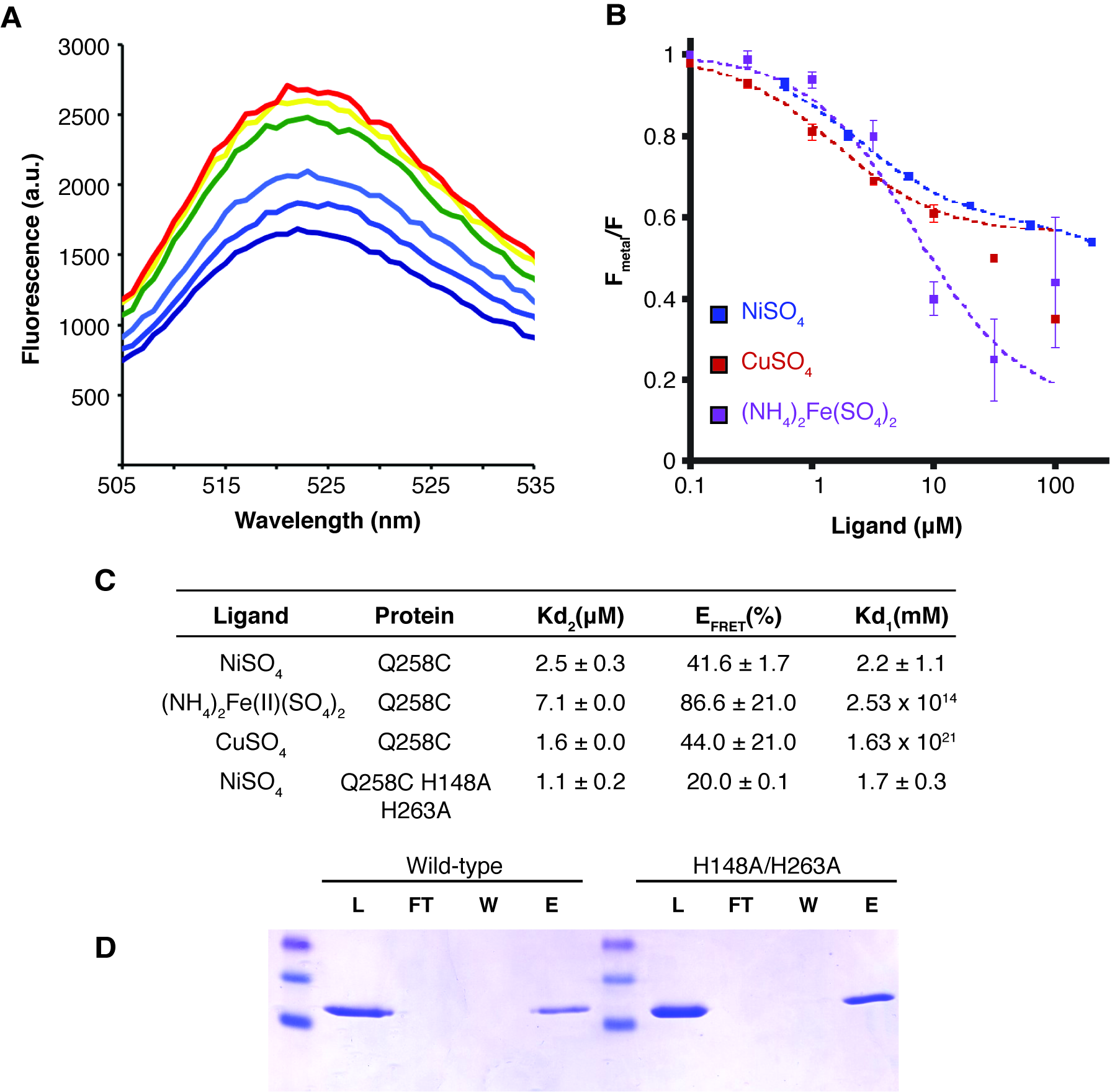

Supplement: S7 Fig — (A) Fluorescence quenching of MamO Q258C labeled with fluorescein-5-maleimide in the presence of increasing concentrations of NiSO4. (B) Binding of various transition metals to labeled MamO. Error bars represent the standard deviation from four independent measurements. The dotted lines are fits to the binding equation described in Methods. (C) Binding constants from tmFRET experiments. (D) Ni-NTA affinity assays with purified protease domains. (TIF) [file pbio.1002402.s009.tif]

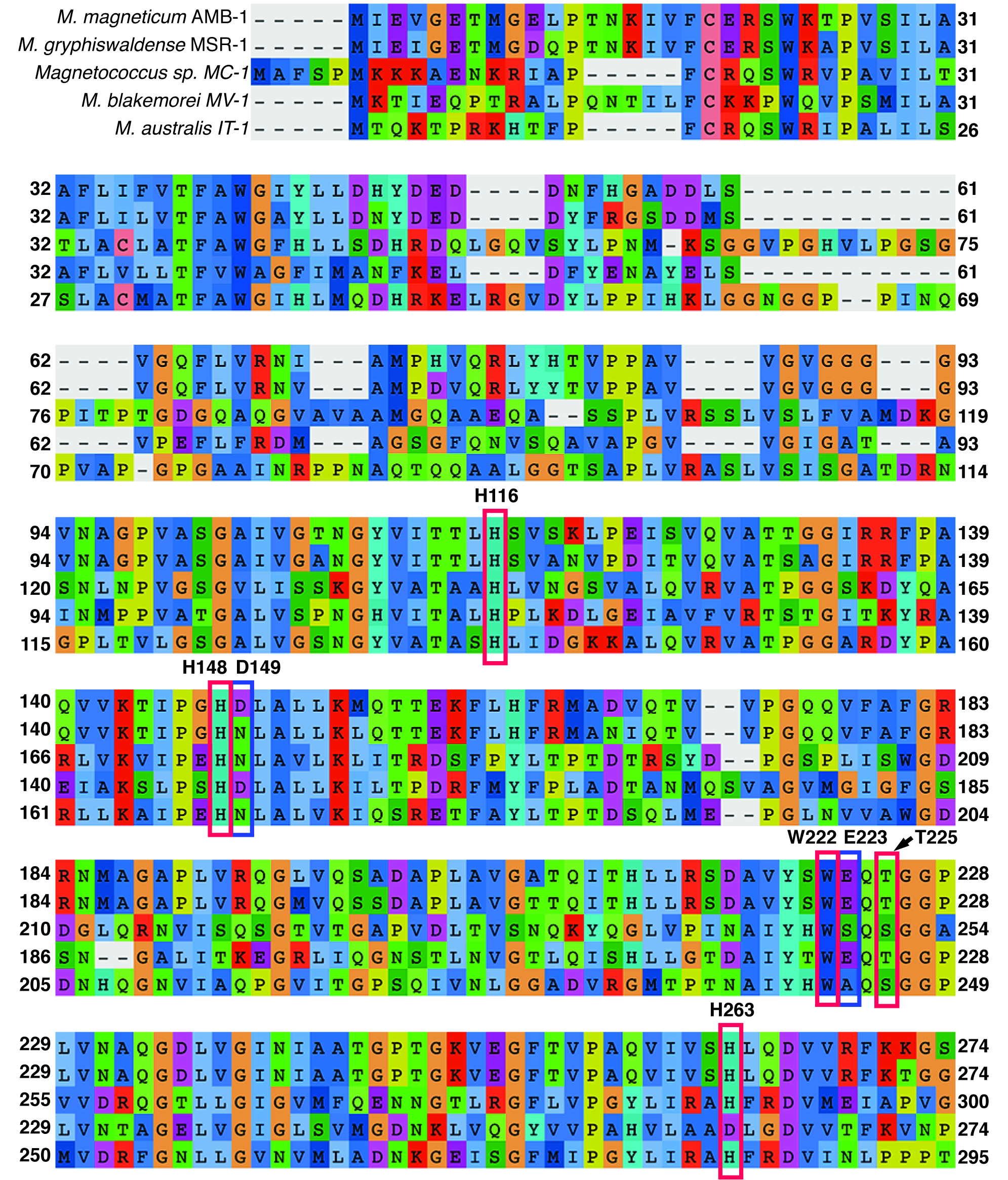

Supplement: S8 Fig — The conservation of critical residues discussed in the text is indicated with colored boxes. (TIF) [file pbio.1002402.s010.tif]

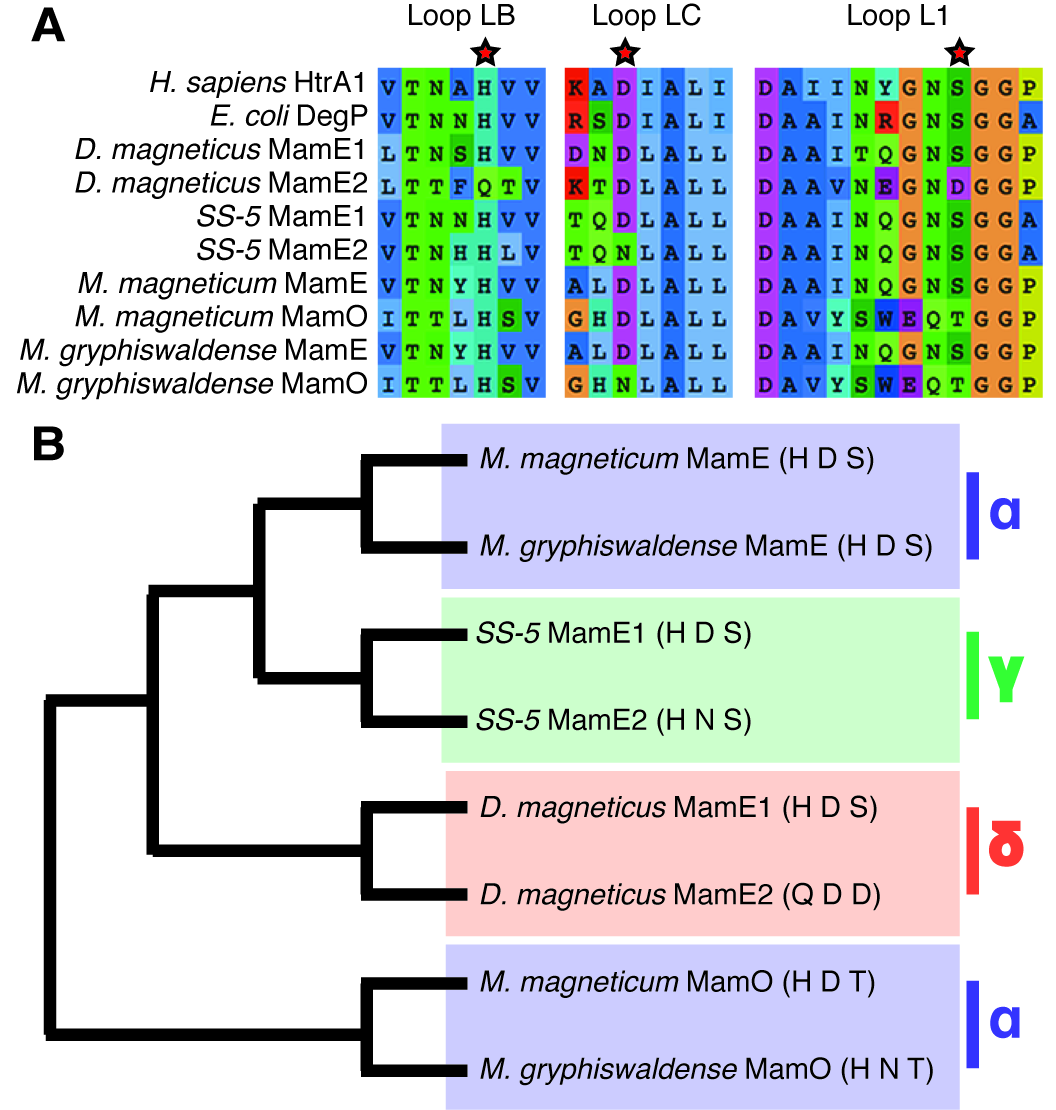

Supplement: S9 Fig — (A) Alignment of the catalytic loops from a set of trypsin-like sequences. The trypsin sequences from four magnetotactic organisms were aligned with two canonical HtrAs, H. sapiens HtrA1 and E. coli DegP. Positions of catalytic triad residues are marked with a star. (B) Phylogeny of the sequences inferred from the detailed analysis shown in Fig 7. The identities of the catalytic triad residues are shown in parentheses after each protein name. Boxes represent the class level taxonomy of the organism within the Proteobacteria. (TIF) [file pbio.1002402.s011.tif]
