# Supplementary material for: MamO Is a Repurposed Serine Protease that Promotes Magnetite Biomineralization through Direct Transition Metal Binding in Magnetotactic Bacteria
Source: PLoS Biol. 2016 Mar 16;14(3):e1002402. doi: 10.1371/journal.pbio.1002402 (PMC4794232; doi:10.1371/journal.pbio.1002402)
Supplement: S1 Table — (DOCX) [file pbio.1002402.s012.docx]

|  | **MamO (apo): 5HM9** | **MamO (Ni): 5HMA** |
| --- | --- | --- |
| Beamline | 8.3.1 at ALS | 8.3.1 at ALS |
| Data collection |  |  |
| Space Group | P432 | P432 |
| Cell Dimensions |  |  |
| *a, b, c* (Å) | 130.22, 130.22, 130.22 | 129.16, 129.16, 129.16 |
| *α, β, γ* (°) | 90, 90, 90 | 90, 90, 90 |
| Wavelength (Å) | 1.116 | 1.116 |
| Resolution (Å) | 50.00-2.60 (2.64-2.60) | 50.00-2.30 (2.34-2.30) |
| No. of Reflections | 12179 | 16999 |
| Completeness (%) | 99.8 (98.5) | 99.9 (100) |
| Redundancy | 15.2 (12.3) | 12.6 (13.1) |
| *R_sym_* (%) | 9.8 (69.5) | 8.5 (89.7) |
| CC_1/2_ (last shell) | 0.577 | 0.905 |
| <I>/<σ(I)> | 79.3 (6.9) | 157.7 (6.3) |
| Refinement |  |  |
| Resolution (Å) | 43.4-2.60 (2.69-2.60) | 43.0-2.30 (2.38-2.30) |
| *R_work_/R_free_* | 20.3/23.2 (27.0/33.0) | 18.5/20.9 (24.4/29.8) |
| TLS Groups | 0 | 3 |
| Average *B*-factor (Å) |  |  |
| Protein | 55.9 | 63.2 |
| Peptide | 77.8 | 98.2 |
| Ni^2+^ (occupancy) |  | 96.0 (1.0) |
| R.m.s deviation from ideality |  |  |
| Bond length (Å) | 0.009 | 0.008 |
| Bond angle (°) | 1.12 | 1.05 |
| Ramachandran Plot |  |  |
| Favored (%) | 96.8 | 97.9 |
| Allowed (%) | 3.2 | 2.1 |
| Outliers (%) | 0 | 0 |

**Table S1** *Summary of crystallographic data*
