# Supplementary material for: MamO Is a Repurposed Serine Protease that Promotes Magnetite Biomineralization through Direct Transition Metal Binding in Magnetotactic Bacteria
Source: PLoS Biol. 2016 Mar 16;14(3):e1002402. doi: 10.1371/journal.pbio.1002402 (PMC4794232; doi:10.1371/journal.pbio.1002402)
Supplement: S2 Table — The main chain configuration at position 193 for all chains in the asymmetric unit of each structure was used to generate the table. (DOCX) [file pbio.1002402.s013.docx]

| **PDB** | **Protein** | **Chain** | **Residue Number (PDB)** | **φ_193_** | **ψ_193_** | **State** | **Reference** |
| --- | --- | --- | --- | --- | --- | --- | --- |
| 3NUM | *H. sapiens* HtrA1 | A | 326 | -65.86 | -33.17 | Inactive | (58) |
| 3NWU | *H. sapiens* HtrA1 | A | 326 | -72.36 | -16.53 | Inactive | (58) |
| 3NWU | *H. sapiens* HtrA1 | B | 326 | -71.58 | -19.3 | Inactive | (58) |
| 3NWU | *H. sapiens* HtrA1 | C | 326 | -70.21 | -18.01 | Inactive | (58) |
| 3MH5 | *E. coli* DegP | A | 208 | -73.41 | -0.05 | Inactive | (59) |
| 3MH5 | *E. coli* DegP | B | 208 | -90.57 | -27.05 | Inactive | (59) |
| 3TJN | *H. sapiens* HtrA1 | A | 326 | -84.24 | -20.3 | Inactive | (60) |
| 3TJN | *H. sapiens* HtrA1 | B | 326 | 137 | 39.78 | Active | (60) |
| 3PV2 | *L. fallonii* DegQ | A | 191 | -48.53 | -38.25 | Inactive | (28) |
| 3PV2 | *L. fallonii* DegQ | B | 191 | -42.8 | -47.42 | Inactive | (28) |
| 3PV2 | *L. fallonii* DegQ | C | 191 | -53.88 | -31.55 | Inactive | (28) |
| 3PV2 | *L. fallonii* DegQ | D | 191 | -56.39 | -41.34 | Inactive | (28) |
| 5HM9 | *M. magneticum* MamO | A | 179 | -73.78 | -10.65 | Inactive | This work |
| 1CGI | *B. taurus* Chymotrypsin | E | 193 | 101.56 | 8.71 | Active | (61) |
| 3PV3 | *L. fallonii* DegQ | A | 191 | 114.28 | 2.93 | Active | (28) |
| 3PV3 | *L. fallonii* DegQ | B | 191 | 119.11 | -35.67 | Active | (28) |
| 3PV3 | *L. fallonii* DegQ | C | 191 | 119.98 | -20.89 | Active | (28) |
| 3PV3 | *L. fallonii* DegQ | D | 191 | 109.9 | -10.84 | Active | (28) |
| 3K6Y | *M. tuberculosis* MarP | A | 341 | 109.05 | -16.85 | Active | (62) |
| 2RCE | *E. coli* DegS | G | 199 | 113.41 | -19.91 | Active | Sauer unpublished |
| 2RCE | *E. coli* DegS | H | 199 | 110.12 | -18.32 | Active | Sauer unpublished |
| 2RCE | *E. coli* DegS | I | 199 | 111.79 | -19.76 | Active | Sauer unpublished |
| 3OTP | *E. coli* DegP | A | 208 | 120.1 | -32.64 | Active | (63) |
| 3OTP | *E. coli* DegP | B | 208 | 118.36 | -30.42 | Active | (63) |
| 3OTP | *E. coli* DegP | C | 208 | 119.61 | -36.86 | Active | (63) |
| 3OTP | *E. coli* DegP | D | 208 | 125.09 | -31.18 | Active | (63) |
| 3OTP | *E. coli* DegP | E | 208 | 121.66 | -33.92 | Active | (63) |
| 3OTP | *E. coli* DegP | F | 208 | 120.96 | -28.68 | Active | (63) |

**Table S2.** *L1 loop conformation in a set of trypsin-like protease structures.* The main chain configuration at position 193 for all chains in the asymmetric unit of each structure was used to generate the table.
