# Supplementary material for: MamO Is a Repurposed Serine Protease that Promotes Magnetite Biomineralization through Direct Transition Metal Binding in Magnetotactic Bacteria
Source: PLoS Biol. 2016 Mar 16;14(3):e1002402. doi: 10.1371/journal.pbio.1002402 (PMC4794232; doi:10.1371/journal.pbio.1002402)
Supplement: S3 Table — The naming in a previous comparative genomic study is unconventional due to confusion over analogy to the MAI in α-Proteobacteria [12]. Our phylogenetic analysis clarifies the ancestry and allows us to use accepted nomenclature. (DOCX) [file pbio.1002402.s014.docx]

| **Lefèvre et al. name** | **Organism** | **Accession** | **Uniprot** | **Triad** | **Suggested name** |
| --- | --- | --- | --- | --- | --- |
| MamEO | RS-1 | YP_002955485 | C4XPP9 | H D S | MamE1 |
| MamE-Nter | RS-1 | YP_002955484 | C4XPP8 | Q D D | MamE2 |
| MamEO | BW-1 | CCO06679 | L0R463 | H D S | MamE1 |
| MamE-Nter-1 | BW-1 | CC06680 | L0R577 | Q D D | MamE2 |
| MamE-Nter-3 | BW-1 | CCO06714 | L0R4A1 | H D S | MamE3 |
| MamE-Nter-2 | BW-1 | CCO06706 | L0R6S4 | H N S | MamE4 |
| MamO | *Ca. Magnetoglobus multicellularis* | ETR64737 | F4ZYU1 | H D S | MamE1 |
| MamE | *Ca. Magnetoglobus multicellularis* | ETR64747 | F4ZYV1 | H N S | MamE2 |
| MamEO-Nter | ML-1 | AFZ77018 | U5IHV7 | H D S | MamE1 |
| MamEO-Cter | ML-1 | AFZ77020 | U5IGM4 | Q D D | MamE2 |
| MamE' | SS-5 | AHY02427 | A0A023UKD7 | H D S | MamE1 |
| MamE | SS-5 | AFX88986 | K7Y606 | H N S | MamE2 |

**Table S3.** *Suggested renaming of the MamE clade.* The naming in a previous comparative genomic study is unconventional due to confusion over analogy to the MAI in *α-Proteobacteria*(12)*.* Our phylogenetic analysis clarifies the ancestry and allows us to use accepted nomenclature.
