# Supplementary material for: MamO Is a Repurposed Serine Protease that Promotes Magnetite Biomineralization through Direct Transition Metal Binding in Magnetotactic Bacteria
Source: PLoS Biol. 2016 Mar 16;14(3):e1002402. doi: 10.1371/journal.pbio.1002402 (PMC4794232; doi:10.1371/journal.pbio.1002402)
Supplement: S4 Table — (DOCX) [file pbio.1002402.s015.docx]

| **Strain** | **Organism** | **Description** | **Source** |
| --- | --- | --- | --- |
| AK30 | *M. magneticum* AMB-1 | Wild-type AMB-1 | (Murat et al. 2010) |
| AK69 | *M. magneticum* AMB-1 | Δ*mamP* | (Murat et al. 2010) |
| AK96 | *M. magneticum* AMB-1 | Δ*mamE* Δ*limE* | (Quinlan et al. 2011) |
| AK94 | *M. magneticum* AMB-1 | Δ*mamO* Δ*R9* | (Quinlan et al. 2011) |
| AK205 | *M. magneticum* AMB-1 | Δ*mamE* Δ*mamO* Δ*R9; mamE* deleted from AK94 using pAK241 | This work |
| AK206 | *M. magneticum* AMB-1 | Δ*mamE* Δ*mamO* Δ*R9 + mamE^WT^;* allele reintroduced with pAK831 | This work |
| AK207 | *M. magneticum* AMB-1 | *ΔmamE ΔmamO* Δ*R9 + mamE^PD^;* allele reintroduced with pAK832 | This work |
| BL21 CodonPlus | *E. coli* | protein expression strain; Cm^R^ | Agilent |
| DH5α (*λpir*) | *E. coli* | standard cloning strain | (Murat et al. 2010) |
| WM3064 | *E. coli* | mating strain; DAP auxotroph used for plasmid transfer | (Murat et al. 2010) |

**Table S4.** *Strains used in this study.*
