# Supplementary material for: MamO Is a Repurposed Serine Protease that Promotes Magnetite Biomineralization through Direct Transition Metal Binding in Magnetotactic Bacteria
Source: PLoS Biol. 2016 Mar 16;14(3):e1002402. doi: 10.1371/journal.pbio.1002402 (PMC4794232; doi:10.1371/journal.pbio.1002402)
Supplement: S5 Table — (DOCX) [file pbio.1002402.s016.docx]

| **Plasmid** | **Description** | **Source** |
| --- | --- | --- |
| pAK241 | Suicide plasmid with fused upsteam and downstream regions to delete *mamE* | (Murat et al. 2010) |
| pAK253 | Plasmid for integration into neutral site in AMB-1 genome; *tac* promoter | (Murat et al. 2010) |
| pAK605 | pAK253 with *tac* promoter replaced by *mamAB* promoter (200bp upstream of *mamH)* | This work |
| pAK619 | pAK605 containing *mamE* | This work |
| pAK620 | pAK605 containing *mamE^PD^* (H188A T211A S297A mutant) | This work |
| pAK831 | pAK241 with the entire *mamE* region inserted, for reintroduction of *mamE* at native locus | This work |
| pAK832 | pAK831 with the S297A mutation | This work |
| pAK845 | pAK605 containing *mamO* | This work |
| pAK856 | pAK845 with the H116A mutation in *mamO* | This work |
| pAK885 | pAK845 with the D149A mutation in *mamO* | This work |
| pAK886 | pAK845 with the T225A mutation in *mamO* | This work |
| pAK887 | pAK845 with the H148A mutation in *mamO* | This work |
| pAK888 | pAK845 with the H263A mutation in *mamO* | This work |
| pAK823 | pAK605 containing M2(3xFLAG)-tagged mamO; the M2 sequence was inserted upstream of *mamO* inframe with a BamHI linker | This work |
| pAK824 | pAK823 with the H116A mutation in *mamO* | This work |
| pAK828 | pAK823 with the D149A mutation in *mamO* | This work |
| pAK825 | pAK823 with the T225A mutation in *mamO* | This work |
| pAK890 | pAK823 with the H148A mutation in *mamO* | This work |
| pAK891 | pAK823 with the H263A mutation in *mamO* | This work |
| pAK876 | MamO protease domain (residues 46-281) inserted into mcsII of pETDuet using NdeI/BglII; for expression of untagged MamOP in E. coli under the control of T7 promoter | This work |
| pAK877 | MamO protease domain (residues 46-281) with GSAWSHPQFEK (strep tag) fused to the C-terminus inserted into mcsII of pETDuet using NdeI/BglII; for expression of MamOP-strep in *E. coli* under the control of T7 promoter | This work |
| pAK889 | pAK877 with Q258C mutation introduced in the MamO protease domain | This work |
| pAK899 | pAK877 with Q258C H148A H263A mutations introduced in the MamO protease domain | This work |

**Table S5.** *Plasmids used in this study.*
